# Attitudes of healthcare professionals towards the use of parenteral methotrexate in rheumatoid arthritis patients: a qualitative study

**DOI:** 10.1007/s11096-026-02183-3

**Published:** 2026-06-30

**Authors:** Jiun Ming Tan, Wern Chern Chai, Nagham Ailabouni, Susanna M. Proudman, Michael D. Wiese, Emily Reeve

**Affiliations:** 1 Centre for Pharmaceutical Innovation, School of Pharmacy and Biomedical Sciences, College of Health, Adelaide University, Adelaide, SA Australia; 2 Health and Biomedical Innovation, School of Pharmacy and Biomedical Sciences, College of Health, Adelaide University, Adelaide, SA Australia; 3 Pharmacy Australia Centre of Excellence, Health and Behavioural Science Department, School of Pharmacy, University of Queensland, Brisbane, QLD Australia; 4 Head of the Rheumatology Unit, Royal Adelaide Hospital, Adelaide, SA Australia; 5 School of Medicine, Adelaide University, Adelaide, Australia; 6 Faculty of Pharmacy and Pharmaceutical Sciences: Centre for Medicine Use and Safety, Monash University, Parkville, VIC Australia

**Keywords:** Methotrexate, Rheumatoid arthritis, Subcutaneous administration, Qualitative research, Healthcare professional attitudes

## Abstract

**Introduction:**

Methotrexate is a cornerstone disease-modifying antirheumatic drug for rheumatoid arthritis (RA), effectively controlling disease activity and reducing cardiovascular mortality. Despite its efficacy, parenteral methotrexate, which offers higher bioavailability, faster response, and fewer gastrointestinal side effects compared to oral administration, remains underutilized in clinical practice. Understanding the factors influencing its utilization is critical to improving RA management.

**Aim:**

To explore the barriers and enablers towards using parenteral methotrexate in rheumatoid arthritis patients from the perspective of Australian healthcare professionals (HCPs).

**Method:**

A qualitative study was conducted using semi-structured interviews between May and August 2022. Participants were recruited via advertising and snowballing. The Theoretical Domains Framework was used to guide the development of the interview guide and data analysis (directed content analysis), supplemented by conventional content analysis (inductive content analysis) to identify emergent themes.

**Results:**

Nineteen HCPs (57.9% male, 42.1% female, 63.2% with more than 10 years of experience) were interviewed, including 14 rheumatologists, 3 pharmacists and 2 nurses. Findings related to domains “environmental context and resources”, “social influences” and “skills” were frequently identified, whereas the domains “intention”, “reinforcement” and “emotion” were least reported. The most prominent enablers related to HCPs' knowledge and skills. Necessary skills were grouped into communication, logistical planning, and patient selection. Other enablers included the accessibility of injection support services, availability of prefilled syringes and online resources for patients (“environmental context and resources”) and social influences. The most common identified barriers related to the logistics of implementing parenteral methotrexate. Other barriers included issues with the Occupational Health and Safety guidelines, time constraints (“environmental context and resources”), concerns about patients withdrawing methotrexate from the vial (“emotion”) and patients’ low acceptability or affordability of the injectable medication (“social influences”).

**Conclusion:**

Our findings suggest that training and guidance on selecting appropriate patients, utilization of systems to support education of patients and to minimize occupational exposure, and availability of patient-friendly devices would support greater implementation of parenteral methotrexate in practice.

**Supplementary Information:**

The online version contains supplementary material available at https://doi.org/10.1007/s11096-026-02183-3.

## Impact statements

- The availability of pre-packed pre-filled syringes has reportedly helped with the implementation of parenteral methotrexate, however, further improvements in the design of the injection device and patient support programs for rheumatoid arthritis patients may be necessary.
- Participants in this study perceived that the influence of consultant rheumatologists was effective in encouraging the utilization of parenteral methotrexate in practice.
- This study highlighted the importance of disseminating scientific evidence pertaining to the benefits of parenteral methotrexate and clarifying that cytotoxic handling precautions are not required for the antirheumatic dose of methotrexate.

## Introduction

Rheumatoid arthritis (RA) is an autoimmune disease for which disease-modifying antirheumatic drugs (DMARDs) are the cornerstone of treatment [1]. Among the available DMARDs, methotrexate is first-line due to its efficacy in controlling the symptoms of RA, low toxicity [2], and benefits in reducing cardiovascular mortality [3]. While oral methotrexate is commonly used in practice, parenteral methotrexate, especially when given via the subcutaneous route, has several advantages over the oral form [4]. Evidence suggests that compared to oral methotrexate in the treatment of RA, subcutaneous methotrexate has higher and more reliable bioavailability, elicits an improved and more rapid response, and is associated with reduced gastrointestinal side effects [5, 6]. Subcutaneous methotrexate may have a cost–benefit advantage to the healthcare system by improving treatment persistence and efficacy of methotrexate, thus potentially delaying the need for the more expensive biologic disease-modifying antirheumatic drugs (bDMARDs) [7–9]. This might be beneficial in developing countries where access to bDMARDs may be limited [9].

Parenteral methotrexate remains underutilized in clinical practice [10]. This was identified in the data from the Australian Pharmaceutical Benefits Scheme (PBS) [11], with similar trends observed in the United States (US) and Spain [10, 12]. Although the reasons for this underutilization are not understood, the attitudes of healthcare professionals (HCPs) towards its use have been reported with other medications [13, 14].

An Australian study found that only one-third of the rheumatologists reported that they trialled parenteral methotrexate in RA patients, but the reasons were not investigated [15]. Further research is needed to understand these findings and to explore the viewpoints of other HCPs. In fact, nurses and pharmacists play a pivotal role in empowering patients to use injectable DMARDs, which is crucial for the implementation of parenteral methotrexate [16, 17].

In Australia, parenteral methotrexate is available in two different forms: vials (the required dose to be drawn up using needles/syringes) and pre-filled syringes. Both are subsidized by the Australian Government through the PBS, but pre-filled syringes are limited for use in patients with psoriasis or severe RA [18]. The pre-filled syringes have only been available since 2017 [19]. Another form of methotrexate injection is the pre-filled pen, which has been widely used in multiple Western countries since 2012 to encourage patient’s self-administration and minimize occupational exposure to methotrexate. This form, however, is not currently available in Australia [20, 21].

To our knowledge, limited qualitative research has specifically explored HCP perspectives on the use of parenteral methotrexate. To address this gap, this study aimed to explore the attitudes of rheumatologists, nurses, and pharmacists towards the use of parenteral methotrexate in RA patients and explore the barriers and enablers to its use.

## Methods

### Study design

A prospective qualitative study using semi-structured one-on-one interviews was conducted. Ethical approvals were obtained from the Central Adelaide Local Health Network (CALHN) Human Research Ethics Committee (CALHN Reference Number: 16084, date of approval: 25 March 2022) and University of South Australia's Human Research Ethics Committee (Application ID: 203438, date of approval: 19 April 2022). This study was reported in accordance with the Standards for Reporting Qualitative Research checklist [22] (Supplementary Material 1).

### Participant selection and recruitment

The inclusion criteria for this study were HCPs practicing in Australia and providing healthcare services to patients with RA, specifically rheumatologists, nurses, and pharmacists. Potential participants were excluded if they were less than 18 years old or not able to read and communicate in English. Recruitment occurred from May to August 2022. HCPs were recruited through an advertisement promoted by the Australian Rheumatology Association (ARA), networks of the investigator group and snowballing. Recruitment was continued until data saturation was achieved, defined as the point at which no new themes or subthemes emerged during the interviews [23].

### Theoretical framework

The theoretical domains framework (TDF) has been extensively used to explore influences on prescribing behaviour and the implementation of healthcare interventions [24–26]. The TDF was used in this study to guide the development of the interview guide and to inform the data analysis. This framework, comprising 14 domains, can be used to identify barriers and facilitators to a desired behaviour in practice [27]. In this study, the behaviours being examined included HCPs’ activities required to support the use of (i.e., implement) parenteral methotrexate in practice including prescribing, dispensing, counselling and administration.

### Interview guide

The interview guide (Supplementary Material 2) contained open- and closed-ended questions as additional prompts where appropriate. All the questions were informed by the TDF and the aims of the study [28]. The interview guide was iteratively revised as necessary throughout the interviews.

### Data collection

Participant demographic information was collected at the beginning of the interview (Table 1), after written consent was obtained. All the interviews were conducted via online video conferencing software (Zoom) or phone with only the audio recorded. Recordings were transcribed using NVivo 12 (QSR International, Doncaster, Australia) software and checked by one investigator (JT).

**Table 1:** Participants’ demographics

Characteristics	Number of participants (%)
*Primary profession*	
Rheumatologist	14 (73.7%)
Pharmacist	3 (15.8%)
Nurse	2 (10.5%)
*Gender*	
Male	11 (57.9%)
Female	8 (42.1%)
*Age group (years)*	
18–30	1 (5.3%)
31–40	4 (21.1%)
41–50	8 (42.1%)
51–60	4 (21.1%)
> 60	2 (10.5%)
*State of Australia practicing in*	
South Australia	8 (42.1%)
New South Wales	4 (21.1%)
Victoria	3 (15.8%)
Queensland	2 (10.5%)
Tasmania	1 (5.3%)
Western Australia	1 (5.3%)
*Location of practice*^a^	
Suburban	11 (57.9%)
City Centre	7 (36.8%)
Regional/remote	4 (21.0%)
*Current practice setting*	
Private	9 (47.4%)
Public	6 (31.6%)
Both private and public	4 (21.1%)
*Years in practice*	
< 5	2 (10.5%)
5–10	5 (26.3%)
11–15	3 (15.8%)
16–20	4 (21.1%)
> 20	5 (26.3%)

^a^Two participants practiced in more than one location

### Data analysis

Data analysis was conducted independently by two researchers (JT and WC) with discussion with other research team members throughout.

Both directed content analysis (DCA, deductive analysis) to the TDF and conventional content analysis (CCA, inductive analysis) were used in accordance with Hsieh and Shannon [29]. This combined approach was chosen because the TDF provided a theory-driven structure to map barriers and enablers to established domains of behaviour change, while CCA allowed new themes to emerge. Relying on DCA alone would have risked overlooking contextual factors not captured by the framework, whereas CCA alone would have lost the ability to link our findings to existing theory.

Data collection and analysis were conducted iteratively. Sample size was guided by the concept of information power [30] and data saturation. Based on the narrow study aim, use of the established TDF and case sampling strategy, a target of 15–30 participants was set (see Supplementary Material 1 for details). In DCA, segments of text (meaning units) were labelled with a code according to the domains listed in the TDF. The researchers then compared the codes and reviewed the definitions of each TDF domain together in order to reach a consensus. Discrepancies were resolved after consultation with a third researcher (ER). The process of reviewing, refining, and applying the coding guidelines was repeated until there were no disagreements. For any meaning units that did not fit existing frameworks as described above, a new code was created (CCA) following the same process as for DCA. All the themes and subthemes generated from this study were then reviewed by the investigator group and amended as necessary.

## Results

A total of 19 participants (11 male; 8 female) comprising 14 rheumatologists, 3 pharmacists and 2 nurses were recruited (Table 1). Participants age was between 41 and 50 years (42.1%), with the majority having more than 10 years of experience (63.2%) in their respective professions. Eighteen interviews were conducted via Zoom, and one was conducted by telephone.

Findings from the interviews were coded into all fourteen TDF domains (Supplementary Material 3). The most commonly identified domains were “environmental context and resources”, “social influences”, and “skills”, while the domains of “intention”, “reinforcement”, and “emotion” were reported less frequently.

### Knowledge

This domain encompassed the knowledge required by HCPs to implement parenteral methotrexate in practice and how an increased awareness of the benefits of parenteral methotrexate can facilitate its uptake in practice.

One key piece of knowledge needed for rheumatologists to implement parenteral methotrexate is when, or in whom, parenteral methotrexate was appropriate. In terms of safety, the use of parenteral methotrexate was reported by participants as being equivalent to the oral route and the risks associated with using parenteral methotrexate were not considered greater than any other injectable medications.

“The risks associated with it [parenteral methotrexate], I would argue, are not any higher than the oral therapy.” **(Pharmacist, private practice, South Australia, 11–15 years of experience).**

For the rheumatologists, being familiar with the available types and formulations of methotrexate injection, along with the relevant PBS guidelines was perceived to facilitate prescribing.

The rheumatologist and nurse participants stated that they and their colleagues possessed the fundamental knowledge and training to manage patients on parenteral methotrexate. On the other hand, the pharmacist participants acknowledged that their peers would require additional education and training to become equally proficient in this regard.

“I think coming out as graduate pharmacists, we don't necessarily know a whole lot. I learn a lot of it on the job and when I was out in practice.”** (Pharmacist, private practice, South Australia, < 5 years of experience).**

Participants reported that more awareness and education around the benefits and safety of parenteral methotrexate were needed to increase use of parenteral methotrexate.

“…I think it [parenteral methotrexate] should be used a lot more than what we do at the moment …More awareness, more data on efficacy and safety.” **(Rheumatologist, public practice, South Australia, 11–15 years of experience).**

### Skills

This domain focused on the practical skills required to support the implementation of parenteral methotrexate. These were grouped into three main areas: communication, logistical planning, and patient selection skills.

Effective communication skills and shared decision-making were considered essential to address patient concerns. Logistical planning skills (e.g., teaching injection technique, needle disposal, and monitoring) developed with experience. Patient selection skills were also important, with suitable candidates including those with intolerable gastrointestinal side effects secondary to oral methotrexate or those not eligible for bDMARDs.

“…I think there's a bit of a learning curve when you go from registrar to consultant in terms of prescribing these things, kind of advising patients on things like sharps container, sharps disposal, injection techniques and things like that…” **(Rheumatologist, public practice, Victoria, < 5 years).**

“So, no, I never force patients. They've got to be comfortable with the plan…” (Rheumatologist, private practice, New South Wales, 5–10 years of experience).

“…Some patients can come with false beliefs about drugs, from limited exposure to discussion about methotrexate. And so you're going to spend a lot of time talking them through the risk benefit ratios of these drugs.” **(Rheumatologist, public practice, Victoria, 5–10 years of experience).**

While participants emphasized the importance of strong communication skills and shared decision-making, a subtle tension emerged. Several rheumatologist participants described “never forcing patients,” yet simultaneously expressed a level of concern with the time required for counselling. These statements suggest that some rheumatologist participants expected patients would proceed with parenteral methotrexate once they had received sufficient information. This shows the tension that can arise between the ideals of shared decision-making and the realities of time-pressured clinical consultations.

### Social influences

This domain described how social influences from peers (within their own profession), other HCPs (of different professions), and patients can affect the implementation of parenteral methotrexate in practice.

Most patients were willing to try methotrexate injections after engaging in a shared decision-making process, although the perception that patients experienced needle phobia remained a barrier for some.

“…there’s been a barrier for anyone who [has] self-administered (injectable) medications, that barrier is rapidly becoming less of a one… there are many more people on injectables for all sorts of things…” **(Rheumatologist, both private and public practice, New South Wales, > 20 years of experience).**

Rheumatologist participants described that patients were generally willing to accept parenteral methotrexate, as the patients liked the benefits of improving disease activity.

“… it’s only once a week, and most of them get used to it, particularly if they feel better from their arthritis…” **(Rheumatologist, private practice, New South Wales, 5–10 years of experience).**

The rheumatologist participants acknowledged that the social influences from their peers or seniors in the clinical setting could have an impact on their decision to use parenteral methotrexate. It was reported that the senior colleagues had stronger influence than peers at the same or more junior level.

“I think that’s part of the reason I feel comfortable doing it [prescribing parenteral methotrexate] is I think I was trained at centres where it was really accepted and safe, it was emphasised and accepted, safe and effective way of escalating treatment. And when you have senior clinicians around you practising in that way, I think that’s probably the biggest way of making a difference…” **(Rheumatologist, public practice, Victoria, < 5 years).**

Regarding the influence of pharmacists and nurses, the rheumatologist participants felt that they had little direct influence on the decision to prescribe parenteral methotrexate. One rheumatologist participant highlighted that the contribution of a pharmacist’s expert medicine advice about prescribing parenteral methotrexate could be useful to them when making prescribing decisions.

### Social/professional role and identity

This domain focused on the roles of all HCPs, as perceived either by the participants themselves or by HCPs from other professions, to promote the safe and effective use of parenteral methotrexate in patients.

Rheumatologist participants perceived injection training as a shared responsibility across professions (GPs, nurses, and pharmacists). Nurses were viewed as administration experts, pharmacists as educators and medicine custodians, and rheumatologists as diagnostic and prescribing experts.

“…the nurse that’s at the GP practice is the one that’s going to end up doing the injections. … And the doctor may be the one that can teach the patient how to do it…… certainly that could be something that can reinforce because I do a really basic [injection training] … And I don’t feel like it’s my role to actually show them how to do it…” **(Rheumatologist, both private and public practice, South Australia, 16–20 years of experience).**

“…nurses are the administration experts …, pharmacists, are the communicators, educators and custodians of the medicine and the medicines information. And then you’ve got prescribers who are the diagnostic experts.” **(Pharmacist, private practice, South Australia, 11–15 years of experience).**

### Beliefs about capabilities

This domain reflected the confidence of the HCP participants to perform their roles in the implementation of parenteral methotrexate. When asked to rate their confidence about their roles, the majority of HCP participants reported high confidence, with the common rating ranging from 7 to 10 (10 being the most confident). The high confidence stemmed from their clinical experience and trust in the evidence.

“…I’m not quite sure that I need support …You have to believe in yourself, and you have to believe in your capacity to pull it off…” **(Rheumatologist, private practice, Tasmania, > 20 years of experience).**

### Beliefs about consequences

This domain represented the beliefs about the outcomes of parenteral methotrexate treatment, whether positive or negative, described by the participants based on their experience. Positive beliefs included its potential to avoid the need for bDMARDs in some patients. Negative beliefs related to limited efficacy or tolerance in certain patients who failed oral methotrexate.

“…So, for some people, they do very well on the parenteral methotrexate, and we don’t need to go to that next step of adding in a biologic [bDMARD]…” **(Rheumatologist, both private and public practice, South Australia, 16–20 years of experience).**

“…I think I always start with oral [methotrexate]. And then you’re only going to use parenteral in patients that don’t tolerate the oral and I just don’t find that there’s that many patients that methotrexate is effective in that don’t tolerate the oral…” **(Rheumatologist, private practice, New South Wales, 5–10 years of experience).**

### Optimism

This domain reflected participants’ optimistic or pessimistic views about the role of parenteral methotrexate.

Optimism attributed to its perceived safety, effectiveness, and affordability. Pessimism was linked to patient burden, needle phobia, and perceptions that it was inferior to bDMARDs in efficacy and device usability.

“… it [methotrexate] forms part of our toolkit. …I think it's very useful in our toolkit. And I think it's probably something that's relatively affordable compared to going straight to a biologic.” **(Rheumatologist, both private and public practice, South Australia, 16–20 years of experience).**

“…it’s mainly from my perspective, the administration burden [of parenteral methotrexate] on the patient… when you’ve got now a biologic option which are also self-injected, injected less frequently and are injected with a better device. … generally, have a greater chance of efficacy.” **(Rheumatologist, private practice, South Australia, 5–10 years of experience).**

### Emotion

This domain captured emotional responses toward parenteral methotrexate use.

Feelings of hopefulness were common when starting treatment. However, some rheumatologist participants expressed worry or discomfort regarding correct patient administration (especially with vials) and communicating with non-English speaking patients via interpreters.

“…I think inherently I wouldn’t start a therapy if I wasn’t at least a little bit hopeful about it in general. Otherwise, it would seem futile,” **(Rheumatologist, public practice, Victoria, 5–10 years of experience).**

“…I’m always very uncomfortable about giving a patient a 50 mg vial and saying draw up 0.4 mL, …, something like that, keep the remaining 25 mg in the fridge until next week. And I just find that whole process a little bit uncomfortable. So, I’d much prefer the pre-filled syringes…” **(Rheumatologist, private practice, South Australia, 5–10 years of experience).**

### Goals

The common treatment goals for patients described by the participants included achieving a remission state or improvement in disease activity with minimal toxicity and reducing disease complications. It was noted, however, that there are alternative treatment options available that can achieve the same goals.

“…the goals of [parenteral methotrexate] treatment really depend on what the patient wants to get out of it… in general terms, I think one restore and maintain functional capacity… The second one is to control symptoms and do that with a good tolerability to the medicines. … the third is longer term as in preventing the long-term disease complications.” **(Rheumatologist, public practice, Queensland, 16–20 years of experience).**

#### Intentions

The concept of “intention” referred to the participants’ decisions to initiate parenteral methotrexate therapy to address specific practice-related issues. One rheumatologist participant reported the tendency to prescribe parenteral methotrexate as a means of avoiding the need for frequent face-to-face consultations, particularly when access to rheumatologists has been limited in the past. The use of parenteral methotrexate was thought to provide more predictable therapeutic outcomes for patients, as it offers better bioavailability, reliable dosage, and reduces the risk of medication errors that may be associated with oral methotrexate.

“There was just only two of us [rheumatologists] and we were just spread so thin that you couldn’t see people easily …From that we moved on to using almost exclusively parenteral methotrexate……” **(Rheumatologist, private practice, Tasmania, > 20 years of experience).**

#### Behavioural regulation

This domain describes how guidelines or research evidence could influence the prescribing of parenteral methotrexate.

The majority of the participants acknowledged that international rheumatology guidelines, such as those published by the European League Against Rheumatism (EULAR) and the American College of Rheumatology (ACR) recommend the use of parenteral methotrexate, which has helped increase awareness and acceptance of parenteral methotrexate in practice.

“… I think the guidelines, ACR and EULAR guidelines regarding rheumatoid… they do generally encourage maximizing the use of methotrexate, including parental methotrexate, before considering biologic therapy…” **(Rheumatologist, both private and public practice, Victoria, > 20 years of experience).**

#### Memory, attention, and decision processes

This domain describes how the HCPs processed the information related to parenteral methotrexate and applied it to their practice. Most rheumatologist participants reported that they were regularly aware of parenteral methotrexate as an option.

However, a smaller group of HCPs highlighted that with so many options available for treating RA, it did occasionally get forgotten. One participant highlighted that this might have been due to the lack of promotion by the pharmaceutical companies. Comparatively, bDMARDs receive more promotions.

“… It's actually less clear, or even imperative, as to why people should prescribe [parenteral methotrexate] at different points… unlike other things [bDMARD] that the advertising isn't quite as in-your-face, and I think there's nothing there to remind you about it [parenteral methotrexate] all the time.” **(Rheumatologist, public practice, Victoria, 5–10 years of experience).**

#### Reinforcement

This domain describes the positive or negative reinforcement for the HCP participants which influenced the prescribing behaviour of parenteral methotrexate in practice. Some of the rheumatologist participants perceived that the current PBS guidelines for methotrexate pre-filled syringes were excessively restrictive, but similar restrictions were not in place for methotrexate vials. Consequently, RA patients with other autoimmune conditions (e.g., psoriatic arthritis, vasculitis, lupus) were not eligible for PBS-funded methotrexate pre-filled syringes.

“If you followed the letter of the law, the PBS [is] probably a bit overly restrictive, … for the Trexject [pre-filled syringe] formulation, …, it’s on the PBS for two treatment indications. One is rheumatoid arthritis. … But the other one is actually skin psoriasis, not psoriatic arthritis.” **(Rheumatologist, public practice, Queensland, 16–20 years of experience).**

It was suggested by one rheumatologist participant that making parenteral methotrexate a mandatory treatment option before bDMARDs under the PBS could potentially serve as a positive reinforcement.

“…probably the biggest way to change behaviour would be to make Medicare [PBS] mandates, make some that has to be trialled before a biologic [bDMARD]. Not that I would want that to happen, but that would be probably the most effective way to make change…” **(Rheumatologist, private practice, South Australia, 5–10 years of experience).**

While participants generally believed that the methotrexate injection did not pose a significant financial burden for patients, they also recognised that patients may face increased costs if they require assistance from HCPs in administering the injection. The pre-filled syringe form also reportedly incurred higher cost than the vial form for the same number of doses. Such financial burden to patients was perceived as a negative reinforcement.

#### Environmental context and resources

This domain highlights the various logistics, resources, services, and legislation that can act as barriers and enablers to the implementation of parenteral methotrexate.

The availability of resources (e.g., online medicine information) and logistics (e.g., support for supply and administration) was deemed essential for ensuring the safe use of parenteral methotrexate. To facilitate the self-injection process, HCPs must provide significant counselling to patients. Alternatively, if a patient is unable to self-administer, logistical arrangements must be made.

Participants highlighted the importance of practical support for patients, including adequate counselling on self-injection technique, needle disposal, and storage. The availability of a rheumatology clinic nurse or community nursing support was viewed as an enabler, particularly for patients unable to self-administer. The availability of pre-filled methotrexate syringes since 2017 was perceived as an appealing solution to overcome logistical barriers and encourage the consideration of parenteral methotrexate as a viable treatment option.

“…I think it [pre-filled syringe] is a lot more attractive [than the vial form] … I’m used to find telling the patients about getting the vial and only drawing as much [they needed], they [the patients] are getting quite stressed about it and some of them are a little bit reticent… and there's a lot of wastage…” **(Rheumatologist, both private and public practice, Victoria, > 20 years of experience).**

However, some logistical barriers persisted. These included limited access to trained support staff for patient education, inconsistent stock availability, safe sharps disposal services, and time constraints during consultations. Time pressure was frequently mentioned by rheumatologists, who noted that thorough education and planning required dedicated time that was often unavailable. Regulatory uncertainty was another reported barrier. Participants noted a lack of clear guidance in Work Health and Safety regulations regarding the handling of low-dose methotrexate, leading some healthcare professionals to apply overly cautious (and unnecessary) precautions.

“…some GP clinic nurses follow the occupational health and Safety Regulations, which are completely nonsense and not science based. … That’s what the barrier is with a lot of community nurse providers” **(Rheumatologist, public practice, Queensland, 16–20 years of experience).**

## Discussion

To our knowledge, this study is the first qualitative study to explore the attitudes of HCPs towards the use of parenteral methotrexate in practice for patients with RA. We used the TDF to identify factors that could facilitate or hinder the implementation of parenteral methotrexate in practice. Findings related to “environmental context and resources”, “social influences” and “skills” were frequently identified, whereas the domains “intention”, “reinforcement” and “emotion” were least reported.

This study forms part of a larger program of research investigating the underutilisation of parenteral methotrexate in RA. Our scoping review [31] synthesized the existing literature on patient-reported barriers and enablers to the use of parenteral methotrexate and highlighted a lack of patient focussed qualitative research and a gap of evidence regarding healthcare professional perspectives. We subsequently explored the views of patients with RA in a complementary qualitative study [32], which identified high overall acceptance of parenteral methotrexate among patients, with key enablers including confidence in self-administration and strong healthcare professional support. The current study complements these earlier works by providing insights from the perspectives of rheumatologists, pharmacists, and nurses—the key professionals involved in prescribing, dispensing, and supporting parenteral methotrexate use. Some discrepancies between rheumatologist perceptions of patient attitudes identified in this study (e.g. concerns that patients were needle phobic) were not identified by patients with RA [32], highlighting the importance of considering patient views and HPA perceptions of patient attitudes. Together, these complementary studies offer a more complete understanding of the multi-level barriers and enablers influencing implementation.

While HCP knowledge and skills were consistently identified as prominent enablers to the uptake of parenteral methotrexate, logistical barriers reportedly existed, including limited time to train patients in self-administration and difficulties in arranging injection assistance for patients unable to self-administer. This is consistent with previous international studies which found that limited consultation time [33], access to GPs and nursing services [34] and patient education [35] were barriers to supporting safe medication use in rheumatology. A review [36] found that patient support programs could have a positive impact on patient adherence and clinical outcomes, emphasizing the need for improving the quality and availability of patient support programs for parenteral methotrexate.

We also found that most rheumatologists and other HCPs considered parenteral methotrexate a therapeutic option based on the scientific evidence and guidelines, indicating a willingness to adhere to evidence-based practice [37, 38]. Furthermore, social influences reinforced the rationale for using parenteral methotrexate through training and work experience, with senior colleagues having a stronger influence compared to junior colleagues. This is consistent with the transfer of training theory, suggesting that supervisors have a more positive influence than peers in the transfer of knowledge and skills [39]. However, some rheumatologist participants had observations during practice that were not consistent with the current research findings, leading to the perception that the benefits of parenteral methotrexate are low. This may be attributed to the cognitive load associated with selecting the appropriate medication for the patient, wherein the HCPs’ focus on bDMARDs and targeted synthetic DMARDs might divert attention from parenteral methotrexate. Bypassing the trial of parenteral methotrexate and directly opting for the more expensive bDMARDs in RA patients may impose an economic burden on the healthcare system [8].

This study highlighted the influence of legislation in practice on the implementation of parenteral methotrexate. Cytotoxic labelling of parenteral methotrexate for RA has led to adoption of chemotherapy precautions by some HCPs, instilling fear in patients [40, 41]. The Council of Australian Therapeutic Advisory Group [42] and the British Royal College of Nursing [43] have stated that no chemotherapy precautions are necessary for HCPs administering low-dose methotrexate via pre-filled pen or syringes, and the same applies to RA patients self-administering parenteral methotrexate. Given that all types of methotrexate were classified as hazardous drugs in the US, the pre-filled pen was developed to minimize the handling requirements and promote patient self-administration [44].

There are some limitations in this study. The sample was heavily weighted toward rheumatologists (14/19 participants), with only three pharmacists and two nurses recruited. This distribution reflects the limited number of other HCPs involved in the use of parenteral methotrexate and means the findings of this study predominantly represent prescribing perspectives. The views from pharmacists and nurses, while consistent with those of rheumatologists, are underrepresented. Therefore, claims regarding overall “HCP perspectives” should be interpreted with caution and might not fully capture the broader multidisciplinary context. Future studies should aim for greater representation from pharmacists and nurses, including those working in community settings. The barriers and enablers identified in this study were derived from the perspective of HCPs, and patients have a distinct view that was not captured in this study, however these specific patient expectations have been discussed elsewhere [32]. Since HCPs recruited are practicing in Australia, the findings from this study may not be generalizable to other countries. Data saturation was achieved as no new themes emerged within the 14 domains of the TDF after 16 interviews and additional 3 interviews were conducted to confirm this. In our final interview, new information about patient’s culture and the influence of other HCPs, such as naturopaths and Chinese medicine practitioners, was identified. However, our study aimed to present selected behaviours (prescribing, dispensing, counselling, administering) relevant to the study objective, rather than encompassing the entire spectrum of social influences from different HCPs. This highlights the need for future studies to investigate these aspects further. There may also be other factors related to our target behaviours that influence treatment decisions that were not raised during our study. Our study also has considerable strengths. We encompassed HCPs from all over Australia, providing a snapshot of the views of these HCPs regarding implementation of parenteral methotrexate. The qualitative approach facilitated an in-depth comprehension of attitudes towards the implementation of methotrexate. To our knowledge, this study is the first to apply the TDF to understand the implementation of parenteral methotrexate, which could facilitate future work to design interventions to support implementation via linking with the Behavioral Change Wheel (BCW) framework [45, 46].

### Clinical implications and recommendations for future research

We found several barriers and enablers through the 14 domains of the TDF. Based on the TDF domains, we propose the following strategies to enhance uptake of parenteral methotrexate:

1. Address environmental context and resources by establishing dedicated parenteral methotrexate support pathways. This could include nurse-led injection clinics or pharmacist-led education programs to reduce the time burden on rheumatologists and improve logistical support for patients unable to self-inject.
2. Target social influences and beliefs about consequences by developing peer-led educational outreach (academic detailing) involving senior rheumatologists who have successfully used parenteral methotrexate.
3. Tackle knowledge, beliefs about capabilities, and environmental context through a multifaceted intervention that includes: (a) updated guidance on safe handling of low-dose methotrexate; (b) practical training on pre-filled syringe prescribing and counselling; and (c) review of PBS restrictions to improve access to pre-filled formulations.

There is a need for more dissemination of knowledge and evidence about parenteral methotrexate to all HCPs involved in the care of RA patients. Interventions to support implementation could therefore include education, persuasion and modelling interventions as described in the BCW framework [45].

Scientific evidence [5] was identified as an enabler in this study (i.e. increased efficacy and tolerability compared to oral methotrexate), but the current ACR guideline [47] includes a low-evidence recommendation on swapping from oral methotrexate to the subcutaneous route for RA. Further clinical trials may be required to provide stronger justification for the benefits of parenteral methotrexate. For example, no direct head-to-head comparison between parenteral methotrexate and bDMARDs has been published, although it has been reported that patients may achieve comparable therapeutic responses with bDMARDs and parenteral methotrexate [48]. The pessimism reported by a small number of participants, such as believing that parenteral methotrexate did not have benefits over oral methotrexate, or that its efficacy is inferior to bDMARDs, seemed to be formed based on personal experiences. These individuals may or may not change their beliefs when presented with existing or new evidence.

Our study has identified the value that HCPs place on GPs, pharmacists and nurses in efficient diffusion and implementation of parenteral methotrexate in practice. Furthermore, there is limited qualitative findings on parenteral methotrexate from the perspective of people with RA [31]. Future qualitative research should consider recruiting GPs and GP nurses to investigate their influence on the implementation of parenteral methotrexate, as well as exploring the patients’ pragmatic experience in using parenteral methotrexate.

This qualitative study provided insights into the barriers and enablers to the use of parenteral methotrexate in RA patients as perceived by HCPs. Our study suggested that the pre-filled syringe facilitated the use of parenteral methotrexate. The social influences from senior HCPs, as well as the knowledge and skills of HCPs, were enablers. However, logistical and resource-related constraints impeded the implementation. Further support in the use of parenteral methotrexate for patients may be necessary.

## Supplementary Information

Below is the link to the electronic supplementary material.

Supplementary file1 (DOCX 24 kb)

Supplementary file2 (DOCX 35 kb)

Supplementary file3 (DOCX 81 kb)

## Data Availability

The authors confirm that the data supporting the findings of this study are available within the article and/or its supplementary materials.

## References

[1] Upchurch KS, Kay J. Evolution of treatment for rheumatoid arthritis. Rheumatology (Oxford). 2012;51(suppl_6):vi28–36. 10.1093/rheumatology/kes278. 23221584

[2] Lopez-Olivo MA, Siddhanamatha HR, Shea B, et al. Methotrexate for treating rheumatoid arthritis. Cochrane Database Syst Rev. 2014;2014(6):Cd000957. 10.1002/14651858.CD000957.pub2. 24916606 PMC7047041

[3] Choi HK, Hernán MA, Seeger JD, et al. Methotrexate and mortality in patients with rheumatoid arthritis: a prospective study. Lancet. 2002;359(9313):1173–7. 10.1016/S0140-6736(02)08213-2. 11955534

[4] Bianchi G, Caporali R, Todoerti M, et al. Methotrexate and rheumatoid arthritis: current evidence regarding subcutaneous versus oral routes of administration. Adv Ther. 2016;33(3):369–78. 10.1007/s12325-016-0295-8. 26846283 PMC4833794

[5] Bujor AM, Janjua S, LaValley MP, et al. Comparison of oral versus parenteral methotrexate in the treatment of rheumatoid arthritis: a meta-analysis. PLoS ONE. 2019;14(9):e0221823-e. 10.1371/journal.pone.0221823. 31490947 PMC6731021

[6] Bakker MF, Jacobs JW, Welsing PM, et al. Are switches from oral to subcutaneous methotrexate or addition of ciclosporin to methotrexate useful steps in a tight control treatment strategy for rheumatoid arthritis? A post hoc analysis of the CAMERA study. Ann Rheum Dis. 2010;69(10):1849–52. 10.1136/ard.2009.124065. 20511610

[7] Fitzpatrick R, Scott DG, Keary I. Cost-minimisation analysis of subcutaneous methotrexate versus biologic therapy for the treatment of patients with rheumatoid arthritis who have had an insufficient response or intolerance to oral methotrexate. Clin Rheumatol. 2013;32(11):1605–12. 10.1007/s10067-013-2318-z. 23835658

[8] Lee J, Pelkey R, Gubitosa J, et al. Comparing healthcare costs associated with oral and subcutaneous methotrexate or biologic therapy for rheumatoid arthritis in the United States. Am Health Drug Benefits. 2017;10(1):42–9. 28465768 PMC5394544

[9] Griffin AJ, Erkeller-Yuksel F. Parenteral methotrexate should be given before biological therapy. Rheumatology (Oxford). 2004;43(5):678. 10.1093/rheumatology/keh110. 15103040

[10] Rohr MK, Mikuls TR, Cohen SB, et al. Underuse of methotrexate in the treatment of rheumatoid arthritis: a national analysis of prescribing practices in the US. Arthritis Care Res (Hoboken). 2017;69(6):794–800. 10.1002/acr.23152. 27863180

[11] Ye JA, Sinnathurai P, Pulver L, et al. Patterns of use of b/tsDMARDs and other medications for patients with rheumatoid arthritis: a retrospective study using PBS data. Intern Med J. 2021;51(Suppl 2):23. 10.1111/imj.15302.

[12] Tornero-Molina J, Andreu JL, Martín-Martínez M-A, et al. Methotrexate in patients with rheumatoid arthritis in Spain: subanalysis of the AR excellence project. Reumatol Clín (English Ed). 2019;15(6):338–42. 10.1016/j.reumae.2018.12.008. 29273497

[13] Mas Dalmau G, Sant Arderiu E, Enfedaque Montes MB, et al. Patients’ and physicians’ perceptions and attitudes about oral anticoagulation and atrial fibrillation: a qualitative systematic review. BMC Fam Pract. 2017;18(1):3. 10.1186/s12875-016-0574-0. 28086887 PMC5234257

[14] Bosanac P, Castle DJ. Why are long-acting injectable antipsychotics still underused? BJ Psych Adv. 2015;21(2):98–105. 10.1192/apt.bp.114.013565.

[15] Hains IS, Hill C, Barrett C, et al. What do rheumatologists think and do in the management of inflammatory arthritis: a baseline survey. Intern Med J. 2021;51(Suppl 2):5–52. 10.1111/imj.15302.

[16] Larsson I, Bergman S, Fridlund B, et al. Patients’ independence of a nurse for the administration of subcutaneous anti-TNF therapy: a phenomenographic study. Int J Qual Stud Health Well Being. 2010;5(2):5146. 10.3402/qhw.v5i2.5146. PMC2899748 20616887

[17] Bornstein C, Craig M, Tin D. Practice guidelines for pharmacists: the pharmacological management of rheumatoid arthritis with traditional and biologic disease-modifying antirheumatic drugs. Can Pharm J (Ott). 2014;147(2):97–109. 10.1177/1715163514521377. 24660010 PMC3962059

[18] Australian Government Department of Health. Methotrexate 10 mg tablet, 50 [Internet]. Canberra: Pharmaceutical Benefits Scheme. Available from: https://www.pbs.gov.au/medicine/item/11268C-11275K-11283W-11288D-11295L. Accessed 01 Aug 2023.

[19] The Australian Pharmaceutical Benefits Scheme. Public Summary Document—March 2017 PBAC Meeting [Internet]. 2017. https://www.pbs.gov.au/info/industry/listing/elements/pbac-meetings/psd/2017-03/methotrexate-psd-march-2017. Accessed 01 Aug 2023.

[20] European Medicines Agency. Methotrexate containing medicinal products [Internet]. Amsterdam: European Medicines Agency; 2019 [updated 21.10.2019]. Available from: https://www.ema.europa.eu/en/medicines/human/referrals/methotrexate-containing-medicinal-products. Accessed 03 Aug 2023.

[21] IQVIA Consulting Services. Case studies for value added medicines: unlocking the potential of patient-centric continuous innovation [Internet]. Brussels: Medicines for Europe; 2019 Jan [updated 2019]. Available from: https://www.medicinesforeurope.com/wp-content/uploads/2019/04/IQVIA-MFE_Case-Studies-for-VAMs_Final-Word-Document_vUpdate2019-v3.0.pdf. Accessed 02 Aug 2023.

[22] O’Brien BC, Harris IB, Beckman TJ, et al. Standards for reporting qualitative research: a synthesis of recommendations. Acad Med. 2014;89(9):1245–51. 10.1097/acm.0000000000000388. 24979285

[23] Saunders B, Sim J, Kingstone T, et al. Saturation in qualitative research: exploring its conceptualization and operationalization. Qual Quant. 2018;52(4):1893–907. 10.1007/s11135-017-0574-8. 29937585 PMC5993836

[24] Atkins L, Francis J, Islam R, et al. A guide to using the theoretical domains framework of behaviour change to investigate implementation problems. Implement Sci. 2017;12(1):77. 10.1186/s13012-017-0605-9. 28637486 PMC5480145

[25] Lee JS, Parashar V, Miller JB, et al. Opioid prescribing after curative-intent surgery: a qualitative study using the theoretical domains framework. Ann Surg Oncol. 2018;25(7):1843–51. 10.1245/s10434-018-6466-x. 29637436 PMC5976533

[26] Cadogan CA, Ryan C, Francis JJ, et al. Improving appropriate polypharmacy for older people in primary care: selecting components of an evidence-based intervention to target prescribing and dispensing. Implement Sci. 2015;10(1):161. 10.1186/s13012-015-0349-3. 26573745 PMC4647274

[27] Cowdell F, Dyson J. How is the theoretical domains framework applied to developing health behaviour interventions? A systematic search and narrative synthesis. BMC Public Health. 2019;19(1):1180. 10.1186/s12889-019-7442-5. 31455327 PMC6712870

[28] Jamshed S. Qualitative research method-interviewing and observation. J Basic Clin Pharm. 2014;5(4):87–8. 10.4103/0976-0105.14194229. 25316987 PMC4194943

[29] Hsieh HF, Shannon SE. Three approaches to qualitative content analysis. Qual Health Res. 2005;15(9):1277–88. 10.1177/1049732305276687. 16204405

[30] Malterud K, Siersma VD, Guassora AD. Sample size in qualitative interview studies: guided by information power. Qual Health Res. 2016;26(13):1753–60. 10.1177/1049732315617444. 26613970

[31] Tan JM, Reeve E, Fraser L, et al. The barriers and enablers to the use of parenteral methotrexate in rheumatoid arthritis patients: a scoping review. Arthritis Care Res (Hoboken). 2023. 10.1002/acr.25141. 37128818

[32] Tan JM, Desforges K, Chai WC, Proudman SM, Wiese MD, Reeve E. Perspectives of patients with rheumatoid arthritis towards the use of parenteral methotrexate: a qualitative study. Int J Clin Pharm. 2026;48(2):557–68. 10.1007/s11096-025-02023-w. 41196547 PMC12992423

[33] Voshaar MJH, van den Bemt BJF, van de Laar MAFJ, et al. Healthcare professionals’ perceptions on barriers and facilitators to DMARD use in rheumatoid arthritis. BMC Health Serv Res. 2022;22(1):62. 10.1186/s12913-021-07459-0. 35022034 PMC8756692

[34] Bernatsky S, Feldman D, De Civita M, et al. Optimal care for rheumatoid arthritis: a focus group study. Clin Rheumatol. 2010;29(6):645–57. 10.1007/s10067-010-1383-9. 20127397

[35] Heidari P, Cross W, Weller C, et al. Rheumatologists’ insight into medication adherence in patients with rheumatoid arthritis: a qualitative study. Int J Rheum Dis. 2019;22(9):1695–705. 10.1111/1756-185X.13660. 31322831

[36] Ganguli A, Clewell J, Shillington AC. The impact of patient support programs on adherence, clinical, humanistic, and economic patient outcomes: a targeted systematic review. Patient Prefer Adherence. 2016;10:711–25. 10.2147/ppa.S101175. 27175071 PMC4854257

[37] Bate L, Hutchinson A, Underhill J, et al. How clinical decisions are made. Br J Clin Pharmacol. 2012;74(4):614–20. 10.1111/j.1365-2125.2012.04366.x. 22738381 PMC3477329

[38] Quiros D, Lin S, Larson EL. Attitudes toward practice guidelines among intensive care unit personnel: a cross-sectional anonymous survey. Heart Lung. 2007;36(4):287–97. 10.1016/j.hrtlng.2006.08.005. 17628198 PMC2034210

[39] Blume BD, Ford JK, Baldwin TT, et al. Transfer of training: a meta-analytic review. J Manag. 2010;36(4):1065–105. 10.1177/0149206309352880.

[40] Lim I. Ridiculous things said about methotrexate [Internet]. Sydney: BJC Health; 2013. Available from: https://www.bjchealth.com.au/connected-care/2013/03/ridiculous-things-said-about-methotrexate/. Accessed 03 Aug 2023.

[41] Arnold MH, Bleasel J, Haq I. Nocebo effects in practice: methotrexate myths and misconceptions. Med J Aust. 2016;205(10):440–2. 10.5694/mja16.00965. 27852171

[42] Council of Australian Therapeutic Advisory Groups. Supporting safe practices for low-dose methotrexate: position statement on the use of low-dose methotrexate [Internet]. Darlinghurst (NSW): CATAG; 2020 Oct. Available from: https://catag.org.au/wp-content/uploads/CATAG-Position-Statement-on-the-use-of-low-dose-methotrexate-1-1.pdf. Accessed 03 Aug 2023.

[43] Royal College of Nursing. Administering subcutaneous methotrexate for inflammatory arthritis [Internet]. 4th ed. London: Royal College of Nursing; 2021. (Publication code: 009 675). Available from: https://www.rcn.org.uk/-/media/Royal-College-Of-Nursing/Documents/Publications/2021/October/009-675.pdf. Accessed 03 Aug 2023.

[44] Schiff M, Jaffe J, Freundlich B, et al. New autoinjector technology for the delivery of subcutaneous methotrexate in the treatment of rheumatoid arthritis. Expert Rev Med Devices. 2014;11(5):447–55. 10.1586/17434440.2014.929492. 24934630

[45] Michie S, van Stralen MM, West R. The behaviour change wheel: a new method for characterising and designing behaviour change interventions. Implement Sci. 2011;6(1):42. 10.1186/1748-5908-6-42. 21513547 PMC3096582

[46] Voshaar M, Vriezekolk J, van Dulmen S, et al. Barriers and facilitators to disease-modifying antirheumatic drug use in patients with inflammatory rheumatic diseases: a qualitative theory-based study. BMC Musculoskelet Disord. 2016;17(1):442. 10.1186/s12891-016-1289-z. 27769224 PMC5075197

[47] Fraenkel L, Bathon JM, England BR, et al. 2021 American College of Rheumatology guideline for the treatment of rheumatoid arthritis. Arthritis Care Res. 2021;73(7):924–39. 10.1002/acr.24596. PMC9273041 34101387

[48] Mainman H, McClaren E, Heycock C, et al. When should we use parenteral methotrexate? Clin Rheumatol. 2010;29(10):1093–8. 10.1007/s10067-010-1500-9. 20544244

