# Supplementary material for: Attitudes of healthcare professionals towards the use of parenteral methotrexate in rheumatoid arthritis patients: a qualitative study
Source: Int J Clin Pharm. 2026 Jun 30;48(5):2026–37. doi: 10.1007/s11096-026-02183-3 (PMC13569598; doi:10.1007/s11096-026-02183-3)
Supplement: Supplementary file 1 [file 11096_2026_2183_MOESM1_ESM.docx]

**Title:** Attitudes of healthcare professionals towards the use of Parenteral Methotrexate in Rheumatoid Arthritis Patients: A Qualitative Study.

**Authors**: Jiun Ming Tan, BPharm (Hons)^1^; Dr Wern Chern Chai, BPharm (Hons) PhD^1^; Dr Nagham Ailabouni, BPharm (Hons) PhD^2^; Professor Susanna Proudman, MBBS (Hons), FRACP^3,4^; Associate Professor Michael D Wiese, BPharm, M Clin Pharm, PhD^1^; Dr Emily Reeve, BPharm (Hons) PhD^5,6^.

**Affiliations**: ^1^ University of South Australia (School of Pharmacy and Medical Sciences), Adelaide (South Australia), Australia; ^2^ Pharmacy Australia Centre of Excellence, Health and Behavioural Science Department, School of Pharmacy, University of Queensland, Brisbane (Queensland), Australia; ^3^ Head of the Rheumatology Unit, Royal Adelaide Hospital, Adelaide (South Australia), Australia; ^4^ Discipline of Medicine, University of Adelaide, Adelaide (South Australia), Australia; ^5^ University of South Australia (Clinical and Health Sciences: Quality Use of Medicines and Pharmacy Research Centre), Adelaide (South Australia), Australia; ^6^ Monash University (Faculty of Pharmacy and Pharmaceutical Sciences: Centre for Medicine Use and Safety), Parkville (Victoria), Australia.

**Correspondence**:
Associate Professor Michael D Wiese, BPharm, M Clin Pharm, PhD,
University of South Australia (School of Pharmacy and Medical Sciences), Adelaide (South Australia), Australia.
Michael.Wiese@unisa.edu.au
 **Journal**: International Journal of Clinical Pharmacy.

**Supplementary material:** Standards for Reporting Qualitative Research Checklist.

**Standards for Reporting Qualitative Research (SRQR)**

O’Brien B.C., Harris, I.B., Beckman, T.J., Reed, D.A., & Cook, D.A. (2014). Standards for reporting qualitative research: a synthesis of recommendations. *Academic Medicine, 89(9)*, 1245-1251.

| **No. Topic** | **Item** | **Page number that reported the key element** |
| --- | --- | --- |
| **Title and abstract** |  |  |
| S1 Title | Concise description of the nature and topic of the study identifying the study as qualitative or indicating the approach (e.g., ethnography, grounded theory) or data collection methods (e.g., interview, focus group) is recommended | Please see the “25Sep25_Title_page_HCP_Qualitative_Study” |
| S2 Abstract | Summary of key elements of the study using the abstract format of the intended publication; typically includes objective, methods, results, and conclusions | Please see the “25Sep25_Abstract_HCP_Qualitative_Study” |
| **Introduction** |  |  |
| S3 Problem formulation | Description and significance of the problem/phenomenon studied; review of relevant theory and empirical work; problem statement | Page 1-2 |
| S4 Purpose or research question | Purpose of the study and specific objectives or questions | Page 2 |
| **Methods** |  |  |
| S5 Qualitative approach and research paradigm | Qualitative approach (e.g., ethnography, grounded theory, case study, phenomenology, narrative research) and guiding theory if appropriate; identifying the research paradigm (e.g., positivist, constructivist/interpretivist) is also recommended | Page 2-3 |
| S6 Researcher characteristics and reflexivity | Researchers’ characteristics that may influence the research, including personal attributes, qualifications/experience, relationship with participants, assumptions, or presuppositions; potential or actual interaction between researchers’ characteristics and the research questions, approach, methods, results, or transferability | Prior to the interviews, the researcher (JT) familiarised himself with the interview guide by practicing with another researcher outside of this study. The first two interviews were conducted by the researcher (JT) with pharmacist qualification in Australia under the guidance of the researcher (ER) with experience in qualitative research. Participants were not informed that the interviewer was a pharmacist, only that they were conducting research in this field.  No relationship between the interviewer (JT) and the participants existed before commencement of the study. However, some participants had pre-existing relationships with the members of the research team. This circumstance is unavoidable considering the relatively small size of the rheumatology field in Australia. |
| S7 Context | Setting/site and salient contextual factors; rationale^a^ | Page 2 |
| S8 Sampling strategy | How and why research participants, documents, or events were selected; criteria for deciding when no further sampling was necessary (e.g., sampling saturation); rationale^a^ | The Australian Rheumatology Association was requested to promote this study on their website, and individuals who wished to participate were instructed to contact the researcher (JT). Subsequently, all potential participants were contacted by email. Further participants were recruited through snowballing, as well as the personal contacts of the research team.  In this study, sample size was informed by information power and saturation. Information power considers five factors: the study aim, sample specificity, use of established theory, quality of dialogue, and analysis strategy [1].  Information power was used to inform the desired sample size:   - The aim of this study was narrow, focusing solely on the implementation of parenteral methotrexate in the rheumatoid arthritis population. This study also employed the well-established Theoretical Domain Framework (TDF) as its theoretical foundation. Additionally, a case sampling strategy was utilized. These factors supported needing a smaller sample size. - The sample specificity was moderate. We aimed to include 3 different types of healthcare professionals who may have varying years of experience in their role, and work in different settings. Also, JT was a novice researcher, and he had no prior experience in qualitative studies. To improve the quality of the dialogue, it was necessary for JT to undergo additional interview training prior to conducting the study. These factors supported the need for a larger sample size.   Taken altogether and based on the experience of the research team, the target sample size was 15-30 participants. This also aligns with the recommendation [2] that minimum sample size of 12 is required to reach data saturation for a qualitative study.  During analysis, saturation was achieved when no new concepts were identified within the 14 TDF domains after 16 interviews. (However, in our final interview new information about patient’s culture and the influence of other healthcare professionals, such as naturopaths and Chinese medicine practitioners, was identified) |
| S9 Ethical issues pertaining to human subjects | Documentation of approval by an appropriate ethics review board and participant consent, or explanation for lack thereof; other confidentiality and data security issues | Page 2 |
| S10 Data collection methods | Types of data collected; details of data collection procedures including (as appropriate) start and stop dates of data collection and analysis, iterative process, triangulation of sources/methods, and modification of procedures in response to evolving study findings; rationale^a^ | Page 3 |
| S11 Data collection instruments and technologies | Description of instruments (e.g., interview guides, questionnaires) and devices (e.g., audio recorders) used for data collection; if/how the instrument(s) changed over the course of the study | Page 3 |
| S12 Units of study | Number and relevant characteristics of participants, documents, or events included in the study; level of participation (could be reported in results) | Page 5 and the characteristics of participants are presented in Table 1. |
| S13 Data processing | Methods for processing data prior to and during analysis, including transcription, data entry, data management and security, verification of data integrity, data coding, and anonymization/deidentification of excerpts | All audio data was transcribed verbatim using the software program NVivo 12 (QSR International, Doncaster, Australia) voice to text function by the researcher (JT). The transcriptions were then quality checked by the researcher (JT) to ensure accuracy before proceeding with data analysis. |
| S14 Data analysis | Process by which inferences, themes, etc., were identified and developed, including researchers involved in data analysis; usually references a specific paradigm or approach; rationale^a^ | Page 5 |
| S15 Techniques to enhance trustworthiness | Techniques to enhance trustworthiness and credibility of data analysis (e.g., member checking, audit trail, triangulation); rationale^a^ | Page 5 |
| **Results/Findings** |  |  |
| S16 Synthesis and interpretation | Main findings (e.g., interpretations, inferences, and themes); might include development of a theory or model, or integration with prior research or theory | Page 5-16 |
| S17 Links to empirical data | Evidence (e.g., quotes, field notes, text excerpts, photographs) to substantiate analytic findings | Quotes are presented on page 5-16 of the manuscript. More quotes are available in the Supplementary material 3. |
| **Discussion** |  |  |
| S18 Integration with prior work, implications, transferability, and contribution(s) to the field | Short summary of main findings; explanation of how findings and conclusions connect to, support, elaborate on, or challenge conclusions of earlier scholarship; discussion of scope of application/generalizability; identification of unique contribution(s) to scholarship in a discipline or field | Page 16-19 |
| S19 Limitations | Trustworthiness and limitations of findings | Page 17-18 |
| **Other** |  |  |
| S20 Conflicts of interest | Potential sources of influence or perceived influence on study conduct and conclusions; how these were managed | Page 20 |
| S21 Funding | Sources of funding and other support; role of funders in data collection, interpretation, and reporting | Page 20 |

^a^The rationale should briefly discuss the justification for choosing that theory, approach, method, or technique rather than other options available, the assumptions and limitations implicit in those choices, and how those choices influence study conclusions and transferability. As appropriate, the rationale for several items might be discussed together.

**References**

1.	Malterud K, Siersma VD, Guassora AD. Sample Size in Qualitative Interview Studies: Guided by Information Power. Qual Health Res. 2016;26(13):1753-60. DOI: 10.1177/1049732315617444.

2.	Lambert SD, Loiselle CG. Combining individual interviews and focus groups to enhance data richness. J Adv Nurs. 2008;62(2):228-37. DOI: 10.1111/j.1365-2648.2007.04559.x.
