# Supplementary material for: Attitudes of healthcare professionals towards the use of parenteral methotrexate in rheumatoid arthritis patients: a qualitative study
Source: Int J Clin Pharm. 2026 Jun 30;48(5):2026–37. doi: 10.1007/s11096-026-02183-3 (PMC13569598; doi:10.1007/s11096-026-02183-3)
Supplement: Supplementary file 2 [file 11096_2026_2183_MOESM2_ESM.docx]

**Title:** Attitudes of healthcare professionals towards the use of Parenteral Methotrexate in Rheumatoid Arthritis Patients: A Qualitative Study.

**Authors**: Jiun Ming Tan, BPharm (Hons)^1^; Dr Wern Chern Chai, BPharm (Hons) PhD^1^; Dr Nagham Ailabouni, BPharm (Hons) PhD^2^; Professor Susanna Proudman, MBBS (Hons), FRACP^3,4^; Associate Professor Michael D Wiese, BPharm, M Clin Pharm, PhD^1^; Dr Emily Reeve, BPharm (Hons) PhD^5,6^.

**Affiliations**: ^1^ University of South Australia (School of Pharmacy and Medical Sciences), Adelaide (South Australia), Australia; ^2^ Pharmacy Australia Centre of Excellence, Health and Behavioural Science Department, School of Pharmacy, University of Queensland, Brisbane (Queensland), Australia; ^3^ Head of the Rheumatology Unit, Royal Adelaide Hospital, Adelaide (South Australia), Australia; ^4^ Discipline of Medicine, University of Adelaide, Adelaide (South Australia), Australia; ^5^ University of South Australia (Clinical and Health Sciences: Quality Use of Medicines and Pharmacy Research Centre), Adelaide (South Australia), Australia; ^6^ Monash University (Faculty of Pharmacy and Pharmaceutical Sciences: Centre for Medicine Use and Safety), Parkville (Victoria), Australia.

**Correspondence**:
Associate Professor Michael D Wiese, BPharm, M Clin Pharm, PhD,
University of South Australia (School of Pharmacy and Medical Sciences), Adelaide (South Australia), Australia.
Michael.Wiese@unisa.edu.au
 **Journal**: International Journal of Clinical Pharmacy.

**Supplementary material:** Interview question guide.

**Welcome and introduction.**

Thank you for your willingness to be involved and taking the time to join us. My name is ______________ and assisting me is ____________.

You were provided with a Participant Information Sheet about your involvement in this study.

A few key points that I would like to remind you of. We don’t think that anything that is discussed today will cause you discomfort, having said that, if you find that the questions make you uncomfortable, you have the right to skip that particular question or stop participating in this interview.

The information you provide will only be used for this research project; when we publish or present the results of this study, the information will be presented in such a way that you cannot be identified.

The session will be audio recorded and transcribed for us to be able to do our analysis.

No personal data will be shared with others and the information provided will be analysed anonymously and used confidentially.

Do you have any questions about this study, or your involvement?

*Turn on Recorder.*

The goal of this study is to learn about your opinions, experiences, attitudes and beliefs.

There are no right or wrong answers to questions – just ideas, experiences, and opinions, which are all valuable.

For all of our discussion today we are talking about the use of parenteral methotrexate (MTX) - which includes IV, IM, or SC. And we are also just considering use of methotrexate to treat rheumatoid arthritis (RA). We would like you to think about all the different aspects including prescribing, dispensing, counselling, monitoring and administrating, of parenteral MTX in practice – as appropriate for your role in providing care.

| **TDF Domains** | **Questions** |
| --- | --- |
| **Goals** *Mental representations of outcomes or end states that an individual wants to achieve* | What would you say is the main consideration when prescribing/recommending/considering treatment of RA with MTX?  *Prompts:*   - What is the primary goal when treating RA? |
| **Knowledge**  *An awareness of the existence of something*  **Skills** *An ability or proficiency acquired through practice* | What do you know about parenteral MTX for RA? *(Knowledge)*  *Prompts:*   - Do you know what parenteral forms of MTX are available in Australia? - What are the expected risks and benefits of parenteral MTX? How do these compare to oral MTX? Compared to other DMARDs? - Do you currently prescribe/dispense (or have patients who use) parenteral MTX? - How did your patients go on the parenteral MTX? did it work well? What about side effects? Any other issues? Did it change what you thought about parenteral MTX?   In what clinical situations do you prescribe/use/recommend parenteral MTX? (Or What clinical situations do you think parenteral MTX could be used for? *– if they don’t have prior experience*) *(Skills)*  *Prompts:*   - Any other types of patients or clinical situations? - How is the process of starting or caring for a patient on parenteral MTX different to oral MTX? What about compared to starting them on a biologic? - Was there any particular knowledge or skills that you needed to develop in order to be able to start or care for a patient on parenteral MTX? - So we have been discussing the use of parenteral MTX – what do you think about the different types of parenteral MTX – i.e. IV, SC, IM? |
| **Attention, memory, and decision process**  *The ability to retain information, focus selectively on aspects of the environment and choose between two or more alternative* | There is a lot to consider in the decision of a new treatment plan. Can you talk me through your thought process when deciding to prescribe/recommend parenteral MTX? E.g. the things that you think about?  *Prompts:*   - Do you ever forget that parenteral MTX is an option? |
| **Emotions** *A complex reaction pattern, involving experiential, behavioural, and physiological elements, by which the individual attempts to deal with a personally significant matter or event* | How would you feel about prescribing/recommending/caring for someone on parenteral MTX?  *Prompts:*   - Would you feel hopeful about this strategy? What expectations do you have with regard to efficacy? What about with regard to side effects? |
| **Beliefs about capabilities**  *Acceptance of the truth, reality, or validity about an ability, talent, or facility that a person can put to constructive use* | *If participants discuss about the internal factors, use the following questions/prompts*:  On a scale of one to ten, how would you rate your confidence in prescribing parenteral MTX/discussing the parenteral MTX with your patients/caring someone who is using parenteral MTX? Why is that?  *Prompts:*   - Is this different to how you would rate your confidence about oral MTX? Why do you think that is? What about other DMARDs and biologics? |

| **Beliefs about consequences**  *Acceptance of the truth, reality, or validity about outcomes of a behaviour in a given situation* | When considering prescribing parenteral MTX (or dispensing/caring for a patient on parenteral MTX), what potential concerns do you have? Any potential negative outcomes?  *Prompts:*   - Side effects? - Patients’ ability to use parenteral MTX? – have you had any patients where this was a problem? Are there particular types of patients that you would be concerned about using parenteral MTX for this reason? - Patients’ reaction to using an injection, e.g., dislike of needles, needle phobia? – have you had any patients where this was a problem? - Are there any types of patients that you wouldn’t prescribe/recommend using parenteral MTX in? Why? |
| --- | --- |
| **High level/general question** | What are the barriers/enablers to prescribing/recommending/caring for someone on parenteral MTX? |
| *If participants discuss about the external factors, use the following questions/prompts*: | |
| **Social influences** *Those interpersonal processes that can cause individuals to change their thoughts, feelings, or behaviours* | Outside of patient clinical characteristics, what or who could influence your decision to prescribe or recommend parenteral MTX in general or in relation to a specific patient?  What about if a patient requested to use parenteral MTX?? Has this happened to you?  Could other healthcare professionals influence your prescribing practice with parenteral MTX?  *Prompts:*   - Peers? - Nurses or pharmacists? (or for nurses and pharmacist – specialists or other doctors?) - Could they influence you to prescribe more? Or less? |
| **Social/professional role and identity** | What role do other health care professionals have in ensuring safe and effective use of parenteral MTX? |
| **Behavioural regulation**  *Anything aimed at managing or changing objectively observed or measured actions* | Could new research change your prescribing practice? What would this need to look like?  *Prompts:*   - Are there any local, national or international policies, guidelines or protocols influence your prescribing of parenteral MTX? How? - Are you aware of any barriers related to chemotherapy rules and regulations? |
| **Environmental context and resources** *Any circumstance of a person's situation or environment that discourages or encourages the development of skills and abilities, independence, social competence, and adaptive behaviour* | What logistical support do you and patients need to ensure that parenteral MTX is used safely and effectively?  *Prompts:*   - E.g. local guidelines, access to stock, support for patient education (about administration) or to administer? - Does your workplace have any of these supports? Would you like them to? - Are there any resources outside your workplace that you are familiar with? - Do you think that the current resources (either for healthcare professionals or patients) are sufficient to support use of parenteral MTX? Are any new resources or supports needed? - How do the resources and support available for parenteral MTX compare to those for other DMARDs, e.g. biologics? |
| **High level/general questions** | If we, or others, wanted to increase the use of parenteral MTX in Australia – what would be needed to make this happen?  Do you have any other thoughts, comments or questions about use of parenteral MTX? Anything else you want to say on the topic? |
