# Supplementary material for: Attitudes of healthcare professionals towards the use of parenteral methotrexate in rheumatoid arthritis patients: a qualitative study
Source: Int J Clin Pharm. 2026 Jun 30;48(5):2026–37. doi: 10.1007/s11096-026-02183-3 (PMC13569598; doi:10.1007/s11096-026-02183-3)
Supplement: Supplementary file 3 [file 11096_2026_2183_MOESM3_ESM.docx]

**Title:** Attitudes of healthcare professionals towards the use of Parenteral Methotrexate in Rheumatoid Arthritis Patients: A Qualitative Study.

**Authors**: Jiun Ming Tan, BPharm (Hons)^1^; Dr Wern Chern Chai, BPharm (Hons) PhD^1^; Dr Nagham Ailabouni, BPharm (Hons) PhD^2^; Professor Susanna Proudman, MBBS (Hons), FRACP^3,4^; Associate Professor Michael D Wiese, BPharm, M Clin Pharm, PhD^1^; Dr Emily Reeve, BPharm (Hons) PhD^5,6^.

**Affiliations**: ^1^ University of South Australia (School of Pharmacy and Medical Sciences), Adelaide (South Australia), Australia; ^2^ Pharmacy Australia Centre of Excellence, Health and Behavioural Science Department, School of Pharmacy, University of Queensland, Brisbane (Queensland), Australia; ^3^ Head of the Rheumatology Unit, Royal Adelaide Hospital, Adelaide (South Australia), Australia; ^4^ Discipline of Medicine, University of Adelaide, Adelaide (South Australia), Australia; ^5^ University of South Australia (Clinical and Health Sciences: Quality Use of Medicines and Pharmacy Research Centre), Adelaide (South Australia), Australia; ^6^ Monash University (Faculty of Pharmacy and Pharmaceutical Sciences: Centre for Medicine Use and Safety), Parkville (Victoria), Australia.

**Correspondence**:
Associate Professor Michael D Wiese, BPharm, M Clin Pharm, PhD,
University of South Australia (School of Pharmacy and Medical Sciences), Adelaide (South Australia), Australia.
Michael.Wiese@unisa.edu.au
 **Journal**: International Journal of Clinical Pharmacy.

**Supplementary material:** Final qualitative charting table.

| **Theme** | **Summary** | **Examples** |
| --- | --- | --- |
| **Knowledge** | The source of knowledge about parenteral methotrexate was reported to be published research, media, guidelines, training, and lectures, which have had an influence on its implementation in practice. | “*In fact, the idea of using subcutaneous methotrexate was first suggested to me in listening to a lecture being given by someone at Harvard*.” (**Rheumatologist, private practice, South Australia, 5-10 years of experience)**  “…*And there's been good data about using parenteral methotrexate. It's published. EULAR and ACR, they both said the same thing that parenteral methotrexate works best. It's what you should be using before you go to biological because it gives you the best result*.” **(Rheumatologist, private practice, Tasmania, >20 years of experience)** |
|  | The knowledge of when/in who to use parenteral methotrexate, which was generally reported as patients who had not tolerated oral methotrexate. Some healthcare professionals (HCPs) used parenteral methotrexate because of the increase in efficacy, or patients’ swallowing difficulties and/or gastrointestinal intolerance with oral methotrexate. | “*…we normally start with oral methotrexate. And depending how people tolerate it, so if there's side effects like gastrointestinal…, then we often will add folinic acid same as our folic acid treatments the day after methotrexate. If that doesn't work…, then we can change to subcutaneous methotrexate at that point, so either for side effects or for increased absorption, so for better efficacy…*” **(Rheumatologist, both private and public practice, South Australia, 16-20 years of experience)**  “…*in situations where patients were taking oral methotrexate and experiencing gastrointestinal side effects in particular. Maybe for people with swallowing difficulties. And people with possibly diseases of the gastrointestinal tract as well*.” **(Pharmacist, private practice, New South Wales, 16-20 years of experience)** |
|  | Subcutaneous route was identified as the ideal parenteral route for administering methotrexate. Intramuscular route was perceived as a less favourable option due to the injection pain. In contrast, intravenous route was not generally indicated for the rheumatoid field. | “… *I think that in rheumatoid arthritis, both the intramuscular and SC, IV has no role, that's out of the rheumatology. Intramuscular, I think it is, completely pointless, it's more painful. There's more chance of complications. You get local abscess. I just don't see the point of intramuscular*.” **(Rheumatologist, public practice, South Australia, 11-15 years of experience)**  “…*there's a subcut pre-filled injections. I think the subcut formulation in the vials is still available. I think it can be given IM. I don't even use it in IM, and I haven't ever used it IV. But I think that can be used intravenously for chemotherapeutic type indications. I don't think any rheumatologist used IV*…” **(Rheumatologist, public practice, Victoria, 5-10 years of experience)** |
|  | The risk for the patients in using parenteral methotrexate was reported to be equivalent to any injectable medications, no additional risks with parenteral methotrexate compared to the methotrexate tablets. | “*So the risks of parental methotrexate are not dissimilar from the oral form of methotrexate when we're using the right doses for rheumatoid arthritis.”* **(Rheumatologist, both private and public practice, South Australia, 16-20 years of experience)**  “…*there's the risk of anyone who's using a syringe, bruising, bleeding, these are pretty rare… bruises… it's not uncommon, …bleeding doesn't exist really apart from bruising. And there is very rare chance of an infection if someone is using a very poor technique and hasn't cleaned the skin down. And the other risks are the storage and disposal of a syringe device as they send it to sharps containers, so they are the other risks, but equally, you have risks of having a bottle of tablets at home*.” **(Rheumatologist, both private and public practice, New South Wales, >20 years of experience)** |
|  | Apart from the pharmacology and pharmacokinetic knowledge about methotrexate, which is the basic, the knowledge of how to administer an injection was reported to be important in all types of HCPs (rheumatologists, nurses and pharmacists).    Rheumatologist participants reported that being aware of the types and formulations of methotrexate injection available, as well as the Pharmaceutical Benefit Scheme (PBS) guidelines associated with it, could be useful in prescribing practice.  Pharmacist participants reported that further injection education and training are required as graduate pharmacists were not generally trained for this role.  Nurse participants reported no further knowledge is required as they were trained for this role. | *“… it's always like in prescribing, you need your pharmacology, which is to know that the fact that there is a bioequivalence dose per dose because some things you use a different dose, you're gonna know what doses apply for different delivery methodologies… So you need to just know those characteristics… and how they can be administered. So there's some knowledge, that it's not complex, it's just basic pharmacology.”* **(Rheumatologist, both private and public practice, New South Wales, >20 years of experience)**  “…*I guess you have to have some awareness of how to inject typically if the patient asks you because often they ask you how they do it. You need to have some idea about what the subcut injection is. … it's useful to know sort of what volume they're injecting, so that's why I mentioned the point five mLs rather than one mL, which is what used to be [in the methotrexate injection]…it makes a big difference. You're injecting half of the volume in terms of whether it's going to be uncomfortable or not. And then they stings or not, …. So I think you have to have some knowledge in having prescribed and used and had feedback about what it actually is in real life, some patients are going to ask you, is it going to hurt? How long is it going to take to work? About all of this stuff? So I think that you have to have some knowledge of what thing looks like, how to use it and the response to it*.” **(Rheumatologist, both private and public practice, South Australia, 16-20 years of experience)**  “*Probably just being aware of the formulations, the authority, the PBS codes [of parenteral methotrexate]…*” **(Rheumatologist, public practice, Victoria, <5 years)**  “*From a pharmacist point of view, yes, I had to learn more about the benefit of parenteral administration versus oral and just a general overview of the condition associated with the use of the treatment*.” **(Pharmacist, private practice, New South Wales, 16-20 years of experience)**  “…*I would say knowledge of being able to give a subcut injection… because how can you teach somebody else if you don't know how to do it yourself? Yes, and you have to sort of managing that pre and post injection management and being able to confidently teach somebody what to do. I think we've covered all the other knowledge*.” **(Nurse, public practice, Queensland, >20 years of experience)** |
|  | More dissemination of evidence about parenteral methotrexate was thought to be required to increase the uptake and encourage the implementation. This could be accomplished with more educations to the HCPs. | “*I think they're actual the materials there, and it's actually reasonably good quality. The problem is the dissemination and the uptake……Because it's not really a new drug, it was heavily promoted to doctors, as you would with a new biologic, for instance. That dissemination of what is the published evidence is probably brief for them than what you get for a new med.*” **(Rheumatologist, public practice, Queensland, 16-20 years of experience)**  “*I think more education evidence [about parenteral methotrexate is needed]. And doing that at a registrar level, and so in an early because I think, a lot of practices in terms of the way we practice, a lot of that's built up early and then it's difficult to change once people have established ways of practicing. I think, you see this with pharmaceutical companies as well, they're getting in early with registrars and showing them the evidence and things like that*.” **(Rheumatologist, public practice, Victoria, <5 years)** |
|  |  |  |
| **Skills** | Communication skill was implicitly described as required to increase the uptake of parenteral methotrexate in patients. HCPs with good communication skill will help to debunk the misconception about parenteral methotrexate, reassure the patients about the safety of parenteral methotrexate, reinforce the benefits of parenteral methotrexate, and encourage the patients’ adherence towards parenteral methotrexate. | “*Anyone starts on methotrexate, whether it's oral or parenteral, I give a massive drill and I drum it into them. It's not a cytotoxic, then there I explain the difference between when it's used at higher doses for cancer treatment, and I always warn them that … they're likely to be told the wrong information at some point… Often I get feedback from the patient, oh yes, someone did tell me that time, and they are kind of OK with them because I warned them*.” **(Rheumatologist, private practice, New South Wales, 5-10 years of experience) (debunk the misconception)**  “…*it's all about empowering the patient, giving them the confidence and also to providing the rationale from going from oral to subcut, so to cut down on the GI side effects and things like that. And patients, when they have to hold methotrexate, actually find a resumption of flares. It's almost like a reinforcing that they actually need that methotrexate. So once you get patients on board with it, often then very reluctant to stop it for whatever reason*…” **(Nurse, public practice, South Australia, >20 years of experience) (reinforce and educate the patients)**  “… *If you tell people …this is the best that exists…It [parenteral methotrexate] will lengthen your life. It improves your cardiovascular risk. And look, 80 per cent of people are going to do really well with it. And if it's not for you, then we'll move to something else. So the expectation needs to be that when you're looking at any of these medicines,… give them hope. You need to say this is the world best... This will help you live better*…” **(Rheumatologist, private practice, Tasmania, >20 years of experience) (increase the patient’s willingness to accept the parenteral methotrexate)** |
|  | Effective communication should also involve the patients in the shared-decision process, while respecting the patients’ preference and decision. | “*…we have a big discussion with the patient, but I think you have to be realistic. And if the patient had a massive phobia, we already know that patients don't take their medications as much as they're meant to do. And I think if you add in a significant phobia on how to give the medication, I think you're just increasing the chance of [patients] not going to actually have the drug. And that's not the aim by the end of the day. So, no, I never force patients. They've got to be comfortable with the plan. Sometimes your options are limited. Obviously, you might get to a point where you only have biological,... But no, …I don't push the point too much. They're not going to be comfortable with it.*” **(Rheumatologist, private practice, New South Wales, 5-10 years of experience)**  “…*I think with needle phobia, … it's an issue, but it doesn't mean I wouldn't suggest it. I answer them and getting around that, get them to go and see the GP or the practice nurse or their GP and try and see if we could give it a go and I still try it in those patients, I wouldn't rule it out, but to get around it. But if they are really phobic, I wouldn't. And I don't want to traumatise them…*” **(Rheumatologist, public practice, Victoria, 5-10 years of experience)** |
|  | Logistics arrangement to improve the practicality of the patients in managing the methotrexate injection was reported to be an important skill. This includes educating patients on injection techniques, organising assistance if the patients are not able to perform self-administration, ongoing safety monitoring and sharps managements.  One rheumatologist participant mentioned about the skills to prepare the patients for active involvement in using parenteral methotrexate. This kind of skill requires years of experience to develop as part of the career as a rheumatologist. | “*I think they just need to be set up, so they need to make sure they've got a sharps container and they know how to safely inject. I would usually recommend that my patients who are starting on it, same as any injectable medication booking an appointment with their GP for their first injection so that they can be trained, how to use it, how to use the needle. So if they had to dispose of them safely, sort of, I guess, parenteral specific kind of conversations that we might have with our patients...*” **(Rheumatologist, both private and public practice, South Australia, 16-20 years of experience) (injection techniques and logistics arrangement)**  “…*these little things that you've got to be thinking of, which is, …, sharps containers, where they're going to be stored, …, we put them in, how to get rid of that. And if you can put that in discussion, describe to them how they can do that through pharmacies or hospital pharmacies. And that's easy as well*.” **(Rheumatologist, both private and public practice, New South Wales, >20 years of experience) (logistics and sharps management)**  “…*I think there's a bit of a learning curve when you go from registrar to consultant in terms of prescribing these things, kind of advising patients on things like sharps container, sharps disposal, injection techniques and things like that*…” **(Rheumatologist, public practice, Victoria, <5 years) (injection techniques and sharps management)** |
|  | Risk stratification or patient selection skills are important to select the right types of patients for parenteral methotrexate therapy. | “…*And with initial discussion, I sort of gauged whether they're capable of doing the injections. For those who are not opposed to injections but are not, I guess, whether due to the arthritis or they're not dexterous enough, then that's when I would ask the GP to help administer for them*.” **(Rheumatologist, private practice, Western Australia, 11-15 years of experience)**  “*I wouldn't evaluate them formally. But when you see a patient and you kind of get a general idea as to what's happening…You take it from there*.” **(Rheumatologist, public practice, South Australia, 11-15 years of experience)** |
|  | Circumstances and/or patients’ characteristics that parenteral methotrexate would be most beneficial for, such as when patients showing response to oral methotrexate, but close to remission, or having tolerance issues with oral methotrexate, or not fitting the criteria for biologics therapy, or not able to swallow table or cognitive issues but assistance in administration is available. | “*…the ones who tend to get it [parenteral methotrexate] are those who moderate-to-severe rheumatoid… They've usually had, …, a significant partial response to oral methotrexate. But the dose has been limited by tolerability, so it sort of becomes a second line. [The patient] already demonstrated this work…moderately. If we could just push the [methotrexate] dose up and [the patient] could tolerate, it probably worked very well. So that's why I mean, the patient selection is right…you have to get [right] patient, has to have good understanding of it…*” **(Rheumatologist, public practice, Queensland, 16-20 years of experience)**  “*I think if they were having issues with oral methotrexate and it was perhaps early on, well, if they were having issues with oral, if their rheumatoid had responded really well to methotrexate monotherapy, say they were in remission. And that was a perfectly reasonable thing to continue, that might be the situation or if they were not tolerating oral methotrexate and was very early in the course of the illness. We're a long way away from a biologic, that might be another scenario*.” **(Rheumatologist, private practice, South Australia, 5-10 years of experience)**  “…*if patients are nil by mouth, if they are on peg feeds and things like that or they have a tracheostomy, or if there's a reason why they can't swallow. Perhaps even cognitive issues like someone else in a nursing home might be injecting that methotrexate for them. Yeah, I think there are lots of other reasons*…” **(Nurse, public practice, South Australia, >20 years of experience)** |
|  | Circumstances and/or patients’ characteristics that parenteral methotrexate would NOT be beneficial for mentally, physically, cognitively, logistically incapable, frequent travellers.  Some rheumatologist participants found that parenteral methotrexate might be a hassle and inconvenience for patients who travelling frequently, so this becomes one of the considerations when selecting the patients to start parenteral methotrexate. | “*…sometimes it's difficult when English is not first language, patients got low health literacy, for instance. So you need to have a certain level of health literacy and understanding, most of this … supporting information is in English… the Australian Rheumatology Association has a YouTube to show how you give it. …It often helps them as a resource to say this is the YouTube link to go to. … I don't tend to choose it in in older people who are not sort of like digital savvy, I suppose…*” **(Rheumatologist, public practice, Queensland, 16-20 years of experience) (non-English background, low health literacy, not digital savvy)**  “…*I think people who couldn't physically handle, I think people who aren't, …, really open to the idea, you know that they're going to be non-compliant, you know they are going to find it difficult. It's just all too hard. So, yeah, I think those are the patients that I wouldn't use in*.” **(Rheumatologist, private practice, South Australia, 5-10 years of experience) (not capable to handle injections, not accepting the idea)**  “*Essentially, anyone that's not capable of maintaining compliance, so anyone with cognitive issues [are not suitable to use parenteral methotrexate]. Yeah, those classic red flags are rare. Is the individual capable of self-managing? If they're not self-managing, are they being managed by someone? do the facilities exist for safe delivery? do you have a sufficiently clean environment, they have appropriate storage, appropriate waste management.*” **(Pharmacist, private practice, South Australia, 11–15 years of experience)**  “…*I think the patients that are traveling, that's a big deal to have them because they have to take needles with them and sharp container*…*a lot of patients find that a hassle, which is totally understandable, especially if they're going to go on a plane somewhere.*” **(Rheumatologist, private practice, New South Wales, 5-10 years of experience)**  “*…I suppose the other disadvantage, just in terms of travel, some people get a bit stressed about travelling with needles and needing leashes or, … needing to take, … particularly they're going overseas, being worried about documentation and carrying needles and that sort of stuff which I think, … increasingly as we are using injectable drugs, people are getting bit more used to that*.” **(Rheumatologist, public practice, Victoria, 5-10 years of experience)** |
| **Social influences** | Mixed of patients’ response observed to the administration of parenteral methotrexate, but some participants felt that the prevalence of needle phobia is not that common, and most patients could become willing to try the methotrexate injection with appropriate discussion which was contributed by various factors.  Some participants also thought that the fear of injection is less of an issue nowadays, with many medications come in an injectable form, which in turn might improve the patients’ acceptability. | “…*so needles, most patients are okay if you explain it to them that there's no alternative, but that's not quite true with subcutaneous methotrexate. …But truly new needle phobic patients about one in 50 right, about two percent. … Others are reticent when they go. Is there any other option? … there are other oral options. So patients do push back a little bit saying I'd rather take a tablet before I'll take an injection. But usually it depends on how… amenable the patient is to the treatment… How innovate to try it..*.” **(Rheumatologist, public practice, Queensland, 16-20 years of experience)**  “*it's a little mixed. Some patients develop a real anxiety around injecting themselves and self-injecting, so they become, they get this hyper anxious stage around, even smelling the alcohol swab or looking at the sharps container. So some patients really have a lot of anxiety around it. And other patients report great benefit and this morning, one of our patients, she was more than happy with a subcutaneous methotrexate weekly. They're all a little different. But I've seen both ends of the spectrum*.” **(Nurse, public practice, South Australia, >20 years of experience)**  “…*there's been a barrier for anyone who was self-administered medications, that barrier is rapidly becoming less of a one. I think through things like there are many more people on injectables for all sorts of things from others, you know they are people are familiar with EpiPens more often than before, and we have an injectable for insulins…we got all the semaglutide and then Ozempic preparation there. There are many people had IVF and have had self-administered things. So there's a greater array of people who have had either personally or family members self-administering medications than previous. It's become not so unusual, and therefore they're all also aware that the needles are very fine and very sharp. There is a better community acceptance of that. And so that's easier. And so that removes that sort of initial thing. …there's still those who are a little bit phobic, and you need a little bit of nurturing on the first injection. And then people realize, oh, it wasn't as bad as your imagination. So that's a first hurdle that is psychological..*.” **(Rheumatologist, both private and public practice, New South Wales, >20 years of experience)** |
|  | HCPs perceived that patients’ low acceptability as one of the barriers in implementing the parenteral methotrexate.  Specific reasons for the low acceptability towards parenteral methotrexate were needle phobia, reluctance to take medication due to the RA diagnosis itself, public misconceptions about low-dose methotrexate as cytotoxic agent, difficulty in using or adhering to the treatment due to dexterity issue, affordability issue, and/or the negative experience with oral methotrexate in the past.  The reasons behind the observed patients’ needle phobia were reported to be multifactorial, it might be related to the patients’ cultural beliefs or personal beliefs influenced by the social environment; it might also be contributed by the form that the parenteral methotrexate is available in, which could be intimidating to the patients; and/or it might be due to the local side effect/pain from the injections. | “*…I'm just conditioned now to not offer from the outset just because of the response I've had from patients. Basically the idea of an injection so freakishly as well. Yeah, so it's definitely not my go-to initial thought to offer it*……*Patient fear of needle, that is a thing. … that's really the only big barrier, … I think obviously educated the patient, but I think that's easy between. Normally we have a nurse in a clinic that can do that, but she is on maternity leave at the moment. I can do it. So obviously, …, I think that's the only barrier, providing a right anticipation and patients perception of needles or willingness ……initially when I started, I mostly only offer the oral formulation initially, mostly because from my experience when I offered the choice between injections or tablets, patients never choose injections to begin with…*” **(Rheumatologist, private practice, New South Wales, 5-10 years of experience) (dislike injections, needle phobia)**  *“There is a lower level of understanding around the injectable methotrexate because it was the domain historically of cytotoxic chemotherapy. There's a bit of confusion about, …, as soon as you’re saying an injection and people jump online, they suddenly start getting information about high dose cytotoxic therapies. So, it's that and then I think the reality is that there are a lot of pharmacists who aren't appropriately educated … about how to inject it properly. You've got all the complexities that go with injection as opposed to oral.”* **(Pharmacist, private practice, South Australia, 11–15 years of experience) (public misconception about low-dose methotrexate as cytotoxic agent)**  “*…Of course, it’s a lot to take in when you first got diagnosed rheumatoid and you’ve got the disease itself and all these other things. And then suddenly that you’re faced with, …having to inject yourself. I think it’s a bit of a big step…*” **(Rheumatologist, both private and public practice, South Australia, 16-20 years of experience)** **(RA diagnosis overwhelming)**  “…*the cost of the Trexject prefilled syringes can sometimes be a barrier for patients swapping over to those. So, for example, I had a patient that went over to subcut, she'd been on subcut before, but with the vials and since this is a little while ago. So since the Trexject come through, I said, Well, why don't we do Trexject? So it'll be great. And then when she came back to see me, she thought they were too expensive, so go back to the vials. So which is an interesting aspects. I don't know whether anybody's mentioned that, and I believe it's much more expensive than the tablets. I don't know the cost of the vials versus the tablets, but that's something that I do tend to mention to people, just so they're aware*.” **(Nurse, public practice, Queensland, >20 years of experience) (affordability issue)**  “*And I suppose their pre-conceptions about injections and there might be some cultural, …, for example, I think some sort of races I find are a little bit more suspicious in regards actions and things. Perhaps, some people of Asian background they don't like having blood tests, don't like having needles and things. There’re some cultural beliefs regarding that that might be threatened. Some people have some Mediterranean and European backgrounds. I found similarly can sometimes be a bit more wary of parenteral. They really feel you've upped the ante in terms of danger and things like that… Some patients can come with false beliefs about drugs, from limited exposure to discussion about methotrexate. And so you're going to spend a lot of time talking them through the risk benefit ratios of these drugs*.” **(Rheumatologist, public practice, Victoria, 5-10 years of experience) (patients’ cultural belief)**  “*…I think it's something like sharps management is always an issue … patients getting given a whole sway of plastic or purple plastic to have to manage. That's one way to really annoy patients…*” **(Rheumatologist, public practice, Victoria, 5-10 years of experience) (intimidating to the patients)**  “*I think needle phobia is always, …, one. Sometimes …in terms of the injecting, if they're not needle phobic, but they did not get much fat and stuff like that, painful thighs, or tummy, whatever from doing it or if they're on anticoagulation and they're getting a lot of bruising and stuff, but that doesn't seem to be a major issue with methotrexate*...” **(Rheumatologist, public practice, Victoria, 5-10 years of experience) (local side effect)** |
|  | Rheumatologist participants perceived that patients would be unaccepting is due to the need to potentially have weekly clinic visits – however some participants had found that patients were often comfortable with this approach and adapted to it. | “*…a lot of patients with a once a week are not unhappy to go [to the GP] and do that and have assistance longer term with [methotrexate] injections*.” **(Rheumatologist, both private and public practice, South Australia, 16-20 years of experience)**  “*Some patients don't like giving themselves injections, but it's not really a big deal. Oh, it's only once a week, and most of them get used to it, particularly if they feel better from their arthritis… a lot of the biological agents are injectable, so subcut injection is not a big barrier for rheumatology patients who are doing it..*.” **(Rheumatologist, private practice, New South Wales, 5-10 years of experience)** |
|  | There are circumstances described by the rheumatologist participants that the patients were willing to accept parenteral methotrexate, as they liked the benefits of improving disease activity. | “*…quite a lot of patients don't like the concept of an injection. But when they choose it, they choose it because they like the concept of a more rapid response and a greater likelihood of success… And if they achieve that with the methotrexate, I find they don't want to stop the injections. If I get the rheumatoid arthritis into remission, then I offered them the option of switching back to tablets. And a lot of them say, No, I want to stick with the injections.*” **(Rheumatologist, private practice, South Australia, 5-10 years of experience)**  “*…if they're close to clinical remission and they're not offered as a choice and I always discuss options, I'm about adding in another drug. And most of the time in that situation, patients don't want to add an extra drug, and they prefer just to stay on the same amount and switch to an injection…*” **(Rheumatologist, private practice, New South Wales, 5-10 years of experience)** |
|  | The patients could be an influence towards prescribing parenteral methotrexate, but it was unlikely to happen in practice due to low patient awareness of this treatment option. | “*…I don't recall ever having a patient coming in and demand it [parenteral methotrexate]. So it's not something that I think is on patients radar anyway. And it's not something that's particularly desirable sought after*.” **(Rheumatologist, private practice, South Australia, 5-10 years of experience)**  “*I'm not aware of it because we obviously don't advertise things. We only see referral after they've gone through their specialist. …, I'm not aware of a request to go injectable initiated by a patient*.” **(Pharmacist, private practice, South Australia, 11–15 years of experience)**  “*Not initially, it will 99.9 percent be the clinician recommending it to be parental methotrexate. But I have seen in instances where patients have had to hold it for different reasons, and they're keen to go back on it to resume it because they feel as how it treats their rheumatoid well. So that's where the patients coming from. Probably not so much from the initiating of it.*” **(Nurse, public practice, South Australia, >20 years of experience)** |
|  | Rheumatologist participants acknowledged that the social influences from their peers or seniors in the clinical setting could have an impact on their decision to use parenteral methotrexate. A supportive workplace atmosphere that encourages the use of parenteral methotrexate can empower HCPs to implement parenteral methotrexate in practice. It was reported that the senior colleagues had stronger influence than peers at the same or lower level. Another way that peers could influence prescribing of parenteral methotrexate is through inheritance of patients who are currently taking parenteral methotrexate. | “*I'm sure they [other rheumatologists] already have [influenced on the decision in prescribing parenteral methotrexate] I think we can see each other all the time as a rheumatologist or trained by other rheumatologists, I was trained by people that are very comfortable and very much promoted, parenteral methotrexate. So I think that definitely influenced my practice. I think we're influenced by each other over time, … Yes, absolutely. I can be influenced by my peers*.” **(Rheumatologist, both private and public practice, South Australia, 16-20 years of experience)**  “Interviewer: *… how did your patients go on to parenteral methotrexate?* **Rheumatologist, private practice, South Australia, 5-10 years of experience***: Some [patients] I inherited from older colleagues and there were already on it [parenteral methotrexate]...* *some had issues with oral methotrexate very early. So we went to parenteral and they stayed on it. But in the rheumatoid arthritis space, those would be the main reasons.*”  “*So I think we have to be really proactive about registrar and trainee and early trainee education. Makes it really acceptable. …I think that's part of the reason I feel comfortable doing it is I think I was trained at centres where it was really accepted and safe, it was emphasized and accepted, safe and effective way of escalating treatment. And when you have senior clinicians around you practicing in that way, I think that's probably the biggest way of making a difference*.” **(Rheumatologist, public practice, Victoria, <5 years)**  *“…I think we've been raised about being resourceful at trying to convince patients to go on these drugs [parenteral methotrexate] and stay on them [methotrexate].”* **(Rheumatologist, public practice, Victoria, 5-10 years of experience)** |
|  | Regarding the influence of pharmacists and nurses, rheumatologist participants felt that they had little influence on prescribing of parenteral methotrexate as current paradigm did not support the influence from other types of HCPs happens – however one rheumatologist participant highlighted that the pharmacist could influence dosing/administration if recommendations were given and evidence-based. Such response was in resonance with the response reported by one pharmacist participant. | “*I don't know if I'd be influenced by other health care practitioners, encouraging me to start subcutaneous methotrexate primarily as well because as the way of the paradigm that we're in at the moment. I don't know if there's an obvious place for that would happen before the discussion with the patient would occur. But also, … I don't think that would influence me*.” **(Rheumatologist, public practice, Victoria, 5-10 years of experience)**  “*Because you practice independently, …, it is all guided by own experiences…*” **(Rheumatologist, public practice, South Australia, 11-15 years of experience)**  “Interviewer: *How about other nurses or pharmacists? Will they affect you how often you prescribe the methotrexate injection?* **Rheumatologist, private practice, New South Wales, 5-10 years of experience**: *Pharmacists can be useful in terms of dosing schedules, because what you guys do, that's where your expertise is. So if you get advice from someone about a dosing schedule that makes sense and is evidence based, you have to listen to it from someone that sort of is an expert in the area.*”  “Interviewer*: have you come across any healthcare professionals such as GP or rheumatologist that they disagree with you [about recommendation of parenteral methotrexate]?*  **Pharmacist, private practice, South Australia, 11–15 years of experience***: No, I think early on there was. And look, you provide them with the papers, pretty hard to argue with the evidence*.” |
| **Beliefs about capabilities** | Rheumatologist participants generally had high self-confidence in prescribing parenteral methotrexate, with most of them gave the rating of 7-10 (10 being the most confident). The high confidence stemmed from the experience of using it in practice and the belief in scientific evidence about parenteral methotrexate. However, some rated it lower – usually because they had more experience with prescribing oral methotrexate.  One rheumatologist participant believed that self-confidence and capability are the most important factors in prescribing parenteral methotrexate. | “Interviewer: …*on a scale of one to 10, how would you rate your confidence in prescribing parenteral methotrexate?* **Rheumatologist, public practice, Queensland, 16-20 years of experience**: *I'd rate it as a nine, and in terms, I'd be pretty confident in discussing it. … pros and cons, practical information*.  Interviewer: *And is that different to how you feel about oral methotrexate?*  **Rheumatologist, public practice, Queensland, 16-20 years of experience**: *No, it will be the same.*  Interviewer: *… do you find any difference in relation to other DMARDs or biologics in terms of your confidence around prescribing?*  **Rheumatologist, public practice, Queensland, 16-20 years of experience:** *It would be pretty much the same. I mean, methotrexate is the anchor drug for rheumatoid. It's probably the most commonly prescribed one… It's probably the one you should have the most confidence in there*.” **(High rating, same rating with oral methotrexate)**  “Interviewer: *…the efficacy and the evidence around side effects. And that seems to have been a key factor in your confidence and in in the parental formulation. Is that right?* **Rheumatologist, public practice, Queensland, 16-20 years of experience**: *Yes*.” **(Confidence in the efficacy and side effects are the reasons for high rating)**  “Interviewer: *on a scale of one to 10, how would you rate your confidence in prescribing parenteral methotrexate?* **Rheumatologist, private practice, Western Australia, 11-15 years of experience**: *I'll say fairly, maybe eight.*  Interviewer: *… is this different to how would you rate your confidence about oral methotrexate?*  **Rheumatologist, private practice, Western Australia, 11-15 years of experience**: *I mean, oral form, … would be nine or 10 if you used it for years.*  Interviewer: *What about other DMARDs and biologics? Would that be different?*  **Rheumatologist, private practice, Western Australia, 11-15 years of experience**: *… the biologic DMARDs, I think, will be around 9 or 10, because that's the bulk of our work. It's just that, unfortunately, methotrexate it's one of those drugs that we really need to keep an eye on, because I've just seen a number of patients where they can do well for years and then all suddenly, white cells drop… the liver spikes and whether it's the change in the diet, the alcohol, or who knows what happens, if something happens with the bone marrow of which case, that's when we lose. That's why you need longer vigilance with methotrexate than I guess with even the biologics.”* **(Lower rating than oral methotrexate)**  “*I don't think you need resources; I think you need yourself, you’ve got to step up to the plate. How do you think people who are selling chemotherapy to somebody with who's got cancer? … They [HCPs] say this is the best. This is going to save your life. This has got the best results, its results based...*” **(Rheumatologist, private practice, Tasmania, >20 years of experience)** |
|  | It was reported that pharmacist participants generally had high rating of confidence in providing care to patients using parenteral methotrexate, which is primarily due to the extensive training and experience received at work.  One pharmacist participant reported that all pharmacists meet the requirement to dispense parenteral methotrexate, but there is a knowledge gap to provide comprehensive care to patients using parenteral methotrexate. This requires extra training and practical experience, especially counselling patients on the injection technique. | *“*Interviewer: …*how would you rate your confidence in dispensing the parenteral methotrexate or discussing about the methotrexate injection? Ten being the most confident.*  **Pharmacist, private practice, South Australia, <5 years of experience***: Probably seven or eight. And I was going to say as a graduate pharmacist or if I hadn't been trained, providing the service that I provided with that company, I think I would be down to two, but I might not be aware that I am a two. … I think it's like because I've had experience providing care. I realise the complexity of it, and I realize the clinical questions and where we don't have enough information or where we need to know more, or the pharmacist could upskill more if I was continuing to supply that service.*  Interviewer: *And is this different to how you would rate your confidence about oral methotrexate?*  **Pharmacist, private practice, South Australia, <5 years of experience**: *For me, I think it's similar.*”  *“But they [other pharmacists] could probably meet the minimum viable. I don't doubt that they can supply it safely and effectively, but being aware of the complexities and the benefits of more regular follow up and stuff*.” **(Pharmacist, private practice, South Australia, <5 years of experience)**  *“I think it's an important and really clear area that pharmacies have scope and knowledge and training around that we can easily provide that counselling. And I think we have a long way to go around injection training and education. I think coming out as graduate pharmacists, we don't necessarily know a whole lot. I learn a lot of it on the job and when I was out in practice. But it is an important area of medicine's administration and medicine supply that can be incorporated into our counselling and our clinical care*.” **(Pharmacist, private practice, South Australia, <5 years of experience)** |
|  | Nurse participants reported high rating of confidence in providing care to patients using parenteral methotrexate. The high confidence reported stemmed from the work experience and learning from senior colleagues and rheumatologists. | **Interviewer:** *…coming back to yourself, on a scale of one to 10, how would you rate your confidence in caring someone who is using methotrexate injection?*  **Nurse, public practice, South Australia, >20 years of experience:** *Oh, it's a nine, I think……I'm more than fallible, but I'm fairly confident. I've had a lot of experience with it, and I work with amazing rheumatologists and I've had some incredible, generous mentoring through the years, … enabled me to become more confident in helping patients with methotrexate…*  **Interviewer:** *… is this different to how you would rate your confidence about oral methotrexate?*  **Nurse, public practice, South Australia, >20 years of experience:** *Well, I think I'd be about a nine too.*  ***Interviewer:*** *How about other DMARDs and biologics?*  **Nurse, public practice, South Australia, >20 years of experience:** *So because I see mostly newly diagnosed rheumatoid, my efforts are concentrating on sulfasalazine and hydroxychloroquine and methotrexate as a triple therapy. That's always our first line of treatment. And later on, if they do fail triple therapy, then they go on to a biologic. And so by that point, yes, there is a bit of information around that. But, once they are on their way and then well-educated, I see less of them. So it's the newly diagnosed rheumatoid that I'm very specific about and make it my priority to see those patients in the clinic.* |
| **Beliefs about consequences** | From the results achieved with their patients, most rheumatologist participants believed that the use of parenteral methotrexate is likely to have positive consequences in the patients, including better therapeutic efficacy, more rapid therapeutic response, more gastrointestinal tolerance compared to oral methotrexate.  HCPs reported that parenteral methotrexate resolved side effects observed with oral methotrexate – nausea, vomiting, alopecia. Some also found that improvement in efficacy after changing from oral to parenteral, especially patients with gastrointestinal issues.  HCPs believed that the use of parenteral methotrexate could avoid the early use of biologics in some patients.  One rheumatologist participant also observed better patients’ compliance with the use of parenteral methotrexate compared to the oral methotrexate. | *“…So there's definitely there's one lady, for instance, that literally anything, any tablet she has, she had nausea like any tablet. And she was very excited to have methotrexate subcut injections for that reason. And she's done very well. But a lot of examples of that, it is very effective in that sense.”* **(Rheumatologist, private practice, New South Wales, 5-10 years of experience) (helpful in gastrointestinal side effects)**  *“I got one patient who is on parenteral methotrexate because she's got short gut syndrome and doesn't resolve with oral methotrexate. And so we've got her on parenteral, it's just as the only way to get the drug into her. Most of the time it's tolerability.”* **(Rheumatologist, private practice, New South Wales, 5-10 years of experience) (Improve in efficacy)**  *“…So for some people, they do very well on the parenteral methotrexate, and we don't need to go to that next step of adding in a biologic…”* **(Rheumatologist, both private and public practice, South Australia, 16-20 years of experience)**  *“… improved efficacy and reduced [methotrexate] dose. So …potentially less representations of side effects or adverse effects for the same or improved efficacy, therefore reduced disease burden and potentially reducing the need progress to more expensive or therapies that they're high risk.”* **(Pharmacist, private practice, South Australia, 11–15 years of experience)**  *“The last majority did quite well. I said initially it is a bit of a barrier for them to convert from the tablet to the injection because, like I said, … they've got scared of injection, but once they get the hang of it, compliance is quite good and the retention rates, quite good as well.”* **(Rheumatologist, private practice, Western Australia, 11-15 years of experience)** |
|  | Some rheumatologist participants admitted that there were patients who would not benefit from the parenteral methotrexate therapy in terms of efficacy or tolerance. However, identifying such group of patients required “trial-and-error” approach, because of the difficulty to predict the therapeutic outcome of methotrexate.  One rheumatologist participant was not convinced by the decision of using parenteral methotrexate after patients failed to tolerate the oral methotrexate due to limited success observed in practice. The same rheumatologist participant also perceived that there are other DMARDs better than methotrexate in general. | *“…So in terms of the benefits, when looking for the same thing, it's just we don't really have great predictors of on an individual level of how he's going to respond well to methotrexate and then traditional disease modifiers, and how he's going to require escalation up to biologics…we have no way of telling, …, definitely who's going to fall into that category. So I also have patients who they are on subcut methotrexate on its own, doesn't provide sufficient disease control, and we do end up ever switching to a biological or adding in a biologic…”* **(Rheumatologist, both private and public practice, South Australia, 16-20 years of experience)**  *“…And in most cases, it seems to improve efficacy of the methotrexate, however some of them are not improving, that may well be that the amount of oral that they were absorbing was pretty much similar to what they were going to be getting subcut…”* **(Rheumatologist, both private and public practice, South Australia, 16-20 years of experience)**  *“…I think I always start with oral [methotrexate]. And then you're only going to use parenteral in patients that don't tolerate the oral and I just don't find that there's that many patients that methotrexate is effective in that don't tolerate the oral…”* **(Rheumatologist, private practice, New South Wales, 5-10 years of experience)** |
| **Optimism** | The optimism reported towards parenteral methotrexate originated from its safety, effectiveness, tolerance, and affordability profiles. | *“… it [methotrexate] forms part of our toolkit. …I think it's very useful in our toolkit. And I think it's probably something that's relatively affordable compared to going straight to a biologic.”* **(Rheumatologist, both private and public practice, South Australia, 16-20 years of experience)**  *“…it's [parenteral methotrexate] an excellent drug for rheumatoid arthritis. …it works well and it's cheap, it's effective. It's just these monitories that's probably the baseline.”* **(Rheumatologist, private practice, Western Australia, 11-15 years of experience)**  *“…If we switch from oral to parenteral, I find that that's often just enough to get them over the line. Yeah, that is how I view it…”* **(Rheumatologist, private practice, New South Wales, 5-10 years of experience)** |
|  | Some rheumatologist participants were pessimistic about the role of parenteral methotrexate given the presence of more effective and patient-friendly biologic treatment options available. Based on their personal beliefs, it was perceived that the parenteral methotrexate was unable to be on a par with the biologic therapy in terms of the efficacy, toxicity, and usability of the injection device. One rheumatologist participant perceived that parenteral methotrexate might not be suitable for moderate-to-severe RA. | *“…I guess when you've got now a biologic option which are also self-injected, injected less frequently and are injected with a better device. And, … generally have a greater chance of efficacy. I don't see that, I see that space for parental methotrexate has narrowed quite a bit in that…”* **(Rheumatologist, private practice, South Australia, 5-10 years of experience)**  *“…It doesn't work as well as the biologics, and it's not as well tolerated, …, it's just not as good a drug. The only reason to use it is because it's cheaper. And I don't think that's a good reason to choose something most of the time, most of the time, …pretty patient centered on the person in front of you and doing what's best for them.”* **(Rheumatologist, private practice, New South Wales, 5-10 years of experience)**  *“… I'm not convinced that in someone with moderate disease activity or high disease activity that getting them to subcut methotrexate, it's going to be adequate to control the disease activity.”* **(Rheumatologist, public practice, Victoria, <5 years)** |
| **Emotion** | The feeling of hopefulness was identified in the participants. | *“…usually I'd only be starting it, always hopeful of a good response. … if it is not a good response or to do with something else, it would be a bummer.”* **(Rheumatologist, public practice, Queensland, 16-20 years of experience)** |
|  | One rheumatologist participant also claimed that no specific emotion associated with the prescribing of parenteral methotrexate. | *“I think inherently I wouldn't start a therapy if I wasn't at least a little bit hopeful about it in general. Otherwise, it would seem futile, to be honest. I think that I wouldn't want to do that to my patients…. you could say that with any medicine. I don't think that I have any specific emotion that I would have as that compared to any other medicine.”* **(Rheumatologist, public practice, Victoria, 5-10 years of experience)**  *“…I don't provoke specific emotions. I don't think I'm so emotive when I prescribe.”* **(Rheumatologist, public practice, Victoria, 5-10 years of experience)** |
|  | The most emotion identified was the feeling of worried that patients might not use the methotrexate injection correctly or inappropriate storage of the injection. The feeling of uncomfortable or uneasy was reported the most when thinking about the patients had to draw the dose of methotrexate from the vials on their own. | *“…I'm always very uncomfortable about giving a patient a 50 mg vial and saying draw up 0.4mL, …, something like that, keep the remaining twenty-five in the fridge until next week. And I just find that whole process a little bit uncomfortable. So, I'd much prefer the pre-filled syringes…”* **(Rheumatologist, private practice, South Australia, 5-10 years of experience)**  *“…I don't like the idea of the option to draw it up themselves… [the patient] had to draw it up because as I said, I'm not comfortable...”* **(Rheumatologist, private practice, New South Wales, 5-10 years of experience)** |
|  | One rheumatologist participant admitted that the feeling of uncomfortable in opening the discussion with patients from non-English speaking background, worrying about the discrepancy in the information delivered to the patients by the interpreters. This was explicitly considered as the barrier in prescribing parenteral methotrexate. | *“…So, where I'm working now, there are a lot of people of non-English speaking background who, … private interpreters and stuff, but I feel uncomfortable. … I suppose I feel a little bit uncomfortable educating people about injections or something that's a little bit more complicated if you don't know that the communication is accurate, so that, I suppose, is a barrier…”* **(Rheumatologist, private practice, New South Wales, 5-10 years of experience)** |
| **Goals** | The common treatment goals for the patients described by the HCPs included achieving remission state or improvement in the disease activity, minimal toxicity from the treatment, reducing the disease complications, and improving the quality of life. There are not unique goals of treatment identified with parenteral methotrexate. The thought of parenteral methotrexate could achieve these goals informed their decision to prescribe parenteral methotrexate, but there are other options to achieve these same goals. | *“…the goals of treatment really depend on what the patient wants to get out of it. But in general terms, I think one restore and maintain functional capacity,... The second one is to control symptoms and do that with a good tolerability to the medicines. So, the medicines don't make you too sick for the benefit. And probably the third is longer term as in preventing the long-term disease complications, but also avoiding long term treatment complications. So, the balance there of benefit and risk long term.”* **(Rheumatologist, public practice, Queensland, 16-20 years of experience)**  *“My goals are multifaceted when I treat someone with rheumatoid arthritis. From the patient's perspective, initially, at least their goal. My goal is to achieve their goal, and that is to reduce pain and improve physical functioning and improve quality of life from the patient's perspective. …The second major goal is to treat their underlying inflammatory disease successfully, and so therefore not only address those concerns of the patient, but also to reduce the risk of joint damage and reduce the risk of cardiovascular disease and all the other comorbidities that occur along with chronic inflammation,...”* **(Rheumatologist, private practice, South Australia, 5-10 years of experience)** |
| **Intentions** | Due to the limited accessibility to the rheumatologist in the past, the parenteral methotrexate was preferably implemented by the rheumatologists to avoid the need and urgency of frequent face-to-face consultation. The intentions of using parenteral methotrexate were described to limit the confounding influence on the pharmacokinetics of methotrexate, ensure the reliable and sustainable therapeutic effect of methotrexate, as well as minimising the medication error from oral methotrexate. | *“…The beginnings of the use of parental methotrexate started for us about 30 odd years ago. There were two of us in practice in XXX and there's 500000 people. So you have to get it right and get good outcomes pretty much straight away. We were using predominantly drawn up methotrexate from the 50 mg ampoules, and you would see people do well with oral methotrexate and then all of a sudden it would all fall over. You'd lose control. Typically, it will be over Christmas time, and I guess there were changes in diet, changes in all sorts of things. So we would then move those to parental methotrexate and at the end of the day, just started using it as the first line rather than going to oral. It saved a lot of buggers arising around in time…”* **(Rheumatologist, private practice, Tasmania, >20 years of experience)**  *“…The other thing that we saw was that the pharmaceutical errors that occur, are always the same. The pharmacists will have a bottle of tablets on hand, and they'll have the stickers that the patient's name on it and instructions and other bits and pieces on their other hand, typically they will have the non-dominant hand, will hold the bottle and a basket with the bottles in it, and now look at the stickers and they'll have all the stickers on their fingers, and then they'll put one sticker on the bottle. So, where medication errors occur is when methotrexate tablet strength is swapped from 2.5 to 10mg……And so the way we achieved getting around medication errors that we went to the leucovorin half a tablet on the day after the methotrexate, which was paralleling what happens with the leukaemia therapy with myeloblastic methotrexate. And that meant you were having either a needle and a tablet, or you were having a tablet of methotrexate, and you had a completely different but half a tablet once a week. So it [the use of parenteral methotrexate] basically eliminated all of the medication errors in one hit.”* **(Rheumatologist, private practice, Tasmania, >20 years of experience)**  *“And so that was the genesis of why we started using it [parenteral methotrexate]. There was just only two of us and we were just spread so thin that you couldn't see people easily ... From that moved on to using almost exclusively parenteral methotrexate at inertia, [we] very, very, very rarely use oral anymore, and it's faster, it's better tolerated. We have less problems.”* **(Rheumatologist, private practice, Tasmania, >20 years of experience)** |
| **Behavioural regulation** | Majority of the participants acknowledged that the evidence-based guidelines, such as The European League Against Rheumatism (EULAR) or American College of Rheumatology (ACR) guidelines, have recommended the use of parenteral methotrexate, which has helped increasing the awareness and making the parenteral methotrexate more acceptable in practice.  Majority participants also highlighted the importance of research evidence and described how it affect their decision to use parenteral methotrexate. They described the anticipated changes in the guideline or research to possibly facilitate the implementation of parenteral methotrexate in practice.  One pharmacist participant suggested that the limited use of parenteral methotrexate may be due to entrenched prescribing habits, despite research evidence and clinical rationale support the use of parenteral methotrexate in certain patients. | *“I think all the rheumatology guidelines, …, where you read will always say, and I think reading the National Prescribing Service (NPS) guidelines this morning, they sent through an email. And it always says that …, consider subcut when oral is poorly tolerated or ineffective and those are things, I don't think it's a barrier...”* **(Rheumatologist, private practice, South Australia, 5-10 years of experience)**  *“… I think the guidelines, ACR and EULAR guidelines regarding rheumatoid. I think they certainly do, probably can't quote the way they are publishing things. But I think they do generally encourage maximising the use of methotrexate, including parental methotrexate, before considering biologic therapy. Yes. Definitely supported, … And maintaining patients on methotrexate, while on biologics in certain cases…”* **(Rheumatologist, public practice, Victoria, 5-10 years of experience)**  *“Nothing other than science. … I make my decisions for my patients based on the best available science and what's best for the patient sitting in front of me and I try to do that without any other influence or bias is, if I can. Now we've all got influences and biases, but …, you try and minimise them.”* **(Rheumatologist, private practice, New South Wales, 5-10 years of experience)**  *“… You wouldn't start any other therapy on the basis that it's not the optimal therapy. We start drugs because they're the appropriate drug and we start them at the lowest likely efficacious dose. The analogy, I would suggest, is that they don't go along and start an antibiotic if it is subtherapeutic dose because it's first line. There's no argument. The first line relates to dose and drug. It doesn't relate to delivery method. Delivery method is a function of access. My understanding is in international jurisdictions where the prefilled syringes were much more readily available, the use of injectable methotrexate is much more common… So, I would challenge anyone to provide me with a logical regime that suggests oral methotrexate an appropriate first line in an individual who is capable of taking parenteral.”* **(Pharmacist, private practice, South Australia, 11–15 years of experience)**  *“And I think it also comes back to it's like, when is it appropriate to use insulin therapy in diabetes? Yeah, that's actually got far less to do with the efficacy and more to do with the perception of the difficulty around injecting. Why would you use, in some cases, three agents when you could use one that goes against the basic tenets? Why use three drugs when you [could] use one? Why use three drugs that have a high side effect profile than the one other, it comes down to, …, it's about ingrained behaviours and thought processes, rather than just go with the rationalised therapy.”* **(Pharmacist, private practice, South Australia, 11–15 years of experience)** |
|  | Some participants, who were already positive about use of subcutaneous methotrexate reported that guidelines or evidence did not influence their decision to prescribe it. | *“I'm aware of the Australian living guidelines for rheumatology, for rheumatoid arthritis. Yeah, I think they're very helpful. But my decision to use parenteral methotrexate predated that.”* **(Rheumatologist, private practice, South Australia, 5-10 years of experience)**  *“I feel like I don't need convincing that it's [parenteral methotrexate] effective or safe. …there's always going to be some risks with it the way they are. But I don't feel like there are any, …, major barriers other than the patients are not being adequate or causing side effects. But …, it's always a trial-and-error thing with our drugs, and so you don't know until you try. So I generally try and methotrexate is pretty much always my first go-to drug with someone with inflammatory arthritis. Unless you've got a patient who's pregnant or…, there's a clear contraindication, I'd always try and start with methotrexate if I can. So and if I need to use subcut, I'm quite happy to use it. I don't feel like I need any research to convince me that it's a good drug.”* **(Rheumatologist, public practice, Victoria, 5-10 years of experience)** |
| **Memory, attention, and decision processes** | Most rheumatologist participants reported that they were being aware of parenteral methotrexate as an option, however, a minority highlighted that with so many options for the treatment of RA, it did occasionally get forgotten as an option.  One participant highlighted that this was caused by the fact that there were no drug companies promoting this drug (but there is a lot of promotion for other drugs like the biologics). | *“…I don't think I forget that it's an option. I think that as an option, it's not always the only option or they are necessarily always the best options for everybody…”* **(Rheumatologist, both private and public practice, New South Wales, >20 years of experience)**  *“… I think it's pretty forefront in my mind. I got a lot of patients on it. I think I used to not use it anywhere near as much when I had to use the vials because it was just quite complex to use and there was all this stuff that needs to be able to talk about it, … being dangerous and how you have to dispose of it and washing your hands afterwards and patients really freaked out by the action. And the guidelines weren't very clear. So, I think in the prefilled syringes, there's not that concern about those sort of things, I think they're a lot safer...”* **(Rheumatologist, public practice, Victoria, 5-10 years of experience)**  *“I think when you have so many options, I think it would be [forgotten]. I never forgot that it was an option. I think we all forget that it's an option. You're dealing with so many therapeutic pathways and possibilities for patients on top of dealing with everything else that's going on with the patient in terms of the impact of disease, life, function and things like that. Absolutely. I do forget. But I think. Often the patient will remind me that that's an option, not in terms of them knowing it's an option, but they'll say, …, do I really need a biologic? And are there any other options? And that sometimes, if I do forget, that usually brings it back to me as an option. And there are patients who are, …, terrified of logical therapies but are much happier to use an injectable drug of something that they already own, for example rather than a new drug.”* **(Rheumatologist, public practice, Victoria, <5 years)**  *“… It's actually less even clear imperative as to why people should prescribe [parenteral methotrexate] at different points. And …, unlike other things that the advertising isn't quite as in-your-face, and I think there's nothing there to remind you about it [parenteral methotrexate] all the time. So, I think that also contributes [to the low uptake of parenteral methotrexate in practice] as well.”* **(Rheumatologist, public practice, Victoria, 5-10 years of experience)**  “…*I'm just reflecting on what I've seen come through emails and stuff the last couple of years through NPS medicine wise and other in the clinical education things. And I think that there's been a lot of focus on the biologics recently, but less so on the parenteral methotrexate..*.” **(Pharmacist, private practice, South Australia, <5 years of experience)** |
|  | There was some cognitive burden reported in the decision to providing care to someone using parenteral methotrexate.  One rheumatologist participant questioned about the role of parenteral methotrexate in practice. There are other options, such as biologics were reported as better in achieving the therapeutic goals. | *“Sometimes it's a choice between going to parenteral methotrexate versus switching to another. Conventional DMARD is very variable, very individual.”* **(Rheumatologist, both private and public practice, South Australia, 16-20 years of experience)**  *“I think from a clinician perspective, … Why would I do that [prescribe parenteral methotrexate] if I can go to a biology? …, and there are certain situations you might, but I think the predominant situations where someone's not tolerating methotrexate or was not working, the next logical step is going, let's give them something which is going to be more effective, probably just as well tolerated or better and easier and less frequent to inject. …, or potential oral. I mean, we have oral JAK inhibitor now, which could be an option…”* **(Rheumatologist, private practice, South Australia, 5-10 years of experience)** |
|  | The thinking process when rheumatologist participants prescribing parenteral methotrexate is reported to be the same as oral methotrexate. The participants reported that patients’ safety, disease activity, convenience, and capability to use injection, as well as patient’s understanding about parenteral methotrexate and accessibility to continual supply of injection, were the aspects the participants considered in their thinking process. | *“…I think it would be like any setting anyone on any form of methotrexate. I just would want to make sure that their baseline blood tests are normal. I think I'm usually for the vast majority…, I would have quite fully already gone through all those checks and looked at their renal function. Does it have to be dose reduced in the setting of renal impairment? I would have made sure the blood counts and their liver function test... I would have made sure that had all the pre-screening done in terms of infectious issues and the hepatitis and HIV and all of this sort of longer-term viral type illnesses that we screened for. So often that's already been through my thought process and gone through all of that by the time we get to parenteral [methotrexate] versus oral… but if I hadn't, I would go through all of that just as I would with oral.”* **(Rheumatologist, both private and public practice, South Australia, 16-20 years of experience)**  *“…No drug interaction… we have noted before that we had individuals who were switching from oral to parenteral. And …there was often times … no accounting for dose equivalents. So they would be grown from 20 oral to 20 injectable, even though itself is not problematic, but you need to consider, obviously, they're effectively increasing the dose by 30 percent. Then you've also got the issue of we had people on oral therapy who were noncompliant. They were basically taking almost PRN because of the oral side effects. Then you see them obviously switch [to parenteral], doctor thinks that they're taking 20mg weekly and then the fact that they are taking 20mg every second or third week. But now you've got an injection of 20 or 25 mgs, you've potentially got a risk there. So you do need to have some insight into compliance in order to account for that dose equivalent.”* **(Pharmacist, private practice, South Australia, 11–15 years of experience)**  *“… I would perhaps take a step back and just go over why the methotrexate is being prescribed…So first, I'd ask them how they feel about it. And so if they're really opposed to it and they say there's no way I'm doing this, well, then certainly we'd sit down and we'd have a conversation about why, why are you not doing this? What is it that you're vehemently opposed to and see if we can work through those steps, see if there's anything that they are obstacles that can be overcome?”* **(Nurse, public practice, South Australia, >20 years of experience)** |
| **Reinforcement** | Positive reinforcement – The Pharmaceutical Benefit Scheme (PBS) mandate of using parenteral methotrexate before biologic treatment can be a positive reinforcement. | *“…probably the biggest way to change behaviour would be to make Medicare mandates, make some that has to be trialled before a biologic. Not that I would want that to happen, but that would be probably the most effective way to make a change…”* **(Rheumatologist, private practice, South Australia, 5-10 years of experience)** |
|  | Positive reinforcement – Being more affordable was considered as the reinforcement to the HCPs as well. HCPs generally perceived that the methotrexate injection was affordable to the patients, with the syringe and vial being the more cost-effective option compared to the prefilled syringe, but the cost for the patients might be increased when assistance required from the HCPs in using parenteral methotrexate. | *“…If someone can't do it, there might be a family member who will do it. I try to avoid a GP doing it or nurses because it just seems to be expensive way of doing things if you're involved in that sort of professional labour force.”* **(Rheumatologist, both private and public practice, New South Wales, >20 years of experience)**  *“…particularly the Trexject brand is a bit more expensive, but I think for patients that find that it works for them if they're not too strapped with their funding, most patients don't find that too. And I think in the context of do we put them on other very expensive drugs that are being subsidised, most people are kind of okay because I have to say that I work in a fairly affluent area, ... So I suppose I probably don't see as many patients that are struggling financially as you might in other suburbs… on the whole, my patients don't seem to have a major issue with the cost of it [parenteral methotrexate].”* **(Rheumatologist, public practice, Victoria, 5-10 years of experience)** |
|  | Negative reinforcement – The Pharmaceutical Benefit Scheme (PBS) guidelines being overly restrictive were perceived as the negative reinforcement to the prescribing of methotrexate injection. | *“…it's more logistical barriers in the community, and it's also a little bit about the PBS. If you followed the letter of the law, the PBS probably a bit overly restrictive, for instance……One of the things, which is interesting because it's for the Trexject formulation, for instance, it's on the PBS for two treatment indications. One is rheumatoid arthritis. That's easy. But the other one is actually skin psoriasis. OK, not psoriatic arthritis. But often patients with psoriatic arthritis have skin psoriasis. And sometimes we use it in things, [like] which inflammatory arthritis, which are not at all either of those, such as adult-onset Still's disease, for instance. And then sometimes we have to pretend that that is sero-negative RA. So, what I'm saying is in one way to make it more accessible is on the PBS to have an unrestricted. … sometimes GPs refused to write the continuation scripts, because they'll go, well, this person's got psoriatic arthritis, that's not one of the two indications. So that in some extent causes a little bit of logistics if people are on it...”* **(Rheumatologist, public practice, Queensland, 16-20 years of experience)**  *“The other problems with this are that PBS funding is restricted. …Rheumatoid arthritis and severe psoriasis, which means that my patients with lupus and vasculitis and even psoriatic arthritis, if they don't have severe psoriasis, don't meet the PBS criteria. And I pointed this out to the Pharmaceutical Benefits Advisory Committee, and they said this is what is approved. So that's a real problem. It is limited my access to parenteral methotrexate...”* **(Rheumatologist, private practice, South Australia, 5-10 years of experience)** |
| **Social/professional role and identity** | The roles self-reported by the rheumatologist participants were selecting the suitable patient to initiate parenteral methotrexate therapy, monitoring the improvement in disease activity and toxicity of methotrexate, and instigating changes in the therapy as required. In terms of injection training, majority of rheumatologist participants were willing to provide the basic injection training (i.e., where to inject? how to use the injection?) during the consultation with their patients. However, the rheumatologist participants perceived that general practitioners, pharmacists, and nurses could also have roles in providing further injection training and/or injection assistance to the patients. | *“I think you should be a rheumatologist or someone who's prescribing methotrexate on a regular basis. I personally feel it should be something it should be initiated by the rheumatologist rather than the GP. I think we're trained to know when it is safe to know what the research, what counselling is required. And I think that's sort of the relationship we have set up with our GP colleagues is that we would be the ones to initiate those changes. And then the GP would, …, support some of that prescribing and some of that monitoring and. Part of that management team, but we would be the ones initiating and instigating changes”* **(Rheumatologist, both private and public practice, South Australia, 16-20 years of experience)**  *“…the nurse that's at the GP practice is the one that's going to end up doing the injections … most of the time. And the doctor may be the one that can teach the patient how to do it. Or even the nurse might teach the patient. And then they'll come back the week after and they'll watch the patient do it. Make sure the technique is correct. And … if they're good at that point in time, they can do it at home... I do a really basic [training], …, this is how you inject kind of thing when I tell the patient…And if the nurses or nurse practitioners had more time to actually go through even at the level of the rheumatology clinic, you probably don't have a nurse. So we'll just describe it ourselves … And I don't feel like it's my role to actually show them how to do it. I just feel not right without the actual injection being in front of me. And so, but sometimes we get it brought into clinic by someone's actually due on that day, and then they're like, I'm still going to GP, but can you do it for me? So we will sometimes do that too...”* **(Rheumatologist, both private and public practice, South Australia, 16-20 years of experience)**  *“So I asked the GPs for assistance, and I offer the patients the option of attending my rooms at 8:30 any morning for assistance in injection training. So GPs, the general practice nurses are critical as well. I think the pharmacists could play an important role, but since X Pharmacy have had to close that side of their business, the role of the pharmacists has been reduced… they [the pharmacists from X Pharmacy] were not only involved in preparing it in the cleanroom, but they [X pharmacy] also sent pharmacists out to patients homes for injection training…”* **(Rheumatologist, private practice, South Australia, 5-10 years of experience)** |
|  | The nurse and pharmacist participants agreed with the rheumatologists' roles and explained how they fitted into the process of caring for patients using parenteral methotrexate. | *“…I know that there's a lot of the tools, … around actually making sure that it's the medication is working and you're achieving the right results, you are looking at like the joints. And so they're like some clinical tools that they use and stuff for that, from the rheumatologist perspective…I guess from a pharmacist perspective, the thing that was most prominent in my mind when I was doing the clinical care was how they were tolerating any side effects. And I will say that the medication was working. But I think our role clinically as a pharmacist really came in around looking at those side effects and making sure that they were going okay with the administration of parenteral methotrexate. So actually doing the subcut injection themselves, making sure that they were adequately trained…”* **(Pharmacist, private practice, South Australia, <5 years of experience)**  *“…once the clinician has diagnosed rheumatoid arthritis and prescribes methotrexate mostly subcutaneously with the patients that I see, then it's my job to make sure that all of that information is reiterated to the patient……As a nurse practitioner, I'm not allowed to prescribe methotrexate. So that would mostly be up to the prescribing doctor…”* **(Nurse, public practice, South Australia, >20 years of experience)** |
|  | Although some of the responsibilities of pharmacists and nurses may appear to overlap, such as providing injection technique counselling, monitoring side effects, and communicating concerns with prescribers while offering support and resources to patients, each profession also has unique roles reported. By working together, HCPs from all pertinent disciplines can help in reducing the incidence of medication misadventure associated with parenteral methotrexate. | *“…I think if they [rheumatologists] ask my opinion, then I'd be able to raise some flags and express concerns. But and then I guess ultimately that's their decision provided they prescribe it safely and appropriately…”* **(Pharmacist, private practice, South Australia, <5 years of experience)**  *“If I really did have some deep concerns about it [the patient on parenteral methotrexate] and I would perhaps go back to the clinician and see if there are any other ways we could treat the rheumatoid because if I have any concerns about I'm not using this safely or equipment safely, then we may have to explore another option. So I always need confirmation from the patient that they're comfortable with this and they understand it and the scripts written properly and make sure that the GP's have adequate information. You know the letter of the last visit. What the plan is going forward for doses, things like that. … a lot of infrastructure is put around the patients to keep them safe between …”* **(Nurse, public practice, South Australia, >20 years of experience)**  “…*nurses are the administration experts, say the ones that delivered the product, pharmacists, are the communicators, educators and custodians of the medicine and the medicines information. And then you've got prescribers who are the diagnostic experts. I see one of the primary roles of the pharmacist is upon presentation of a prescription determining is that the most efficacious? Delivery method of the therapy. So … our challenge is every time we say methotrexate script, we should be asking as a profession, is this patient going to get better disease outcomes by switching to a parenteral, is the parenteral an efficacious, appropriate therapy because there are clearly reasons why we don't want to progress with parenteral, but we should be sitting there going, okay, this is the spectrum of access. These are the outcomes that can overlay that access. Which one brings about the best outcome for the patient? Our role is to advocate for the patient outcome on the basis of our knowledge of drug dose, design those things. Whereas I rightly or wrongly think the nurse is about and should be about more the administration. That's what they were trained to do, that is their specialty… I will have done less injections than some of my nurse colleagues. I'm not going to begin to suggest that my knowledge is superior to them, but I do think that my knowledge with regards to drug dose design and efficacy is superior, and therein lies my role*…” **(Pharmacist, private practice, South Australia, 11–15 years of experience)** |
|  |  |  |
| **Environmental context and resources** | Chemotherapy or Occupational Health and Safety guidelines and the logistical issues related to patient use of low-dose parenteral methotrexate indicated for RA were identified as the barriers. Specific logistical concerns included the availability of support for patient education and self-administration, access to injection devices, stock availability, and sharp waste management. | *“…some GP clinic nurses follow the Occupational health and Safety Regulations, which are completely nonsense and not science based. And we'll just have a blanket. No, this is a cytotoxic treatment. We're not giving that. That's what the barrier is with a lot of community nurse providers directly like blue nurses. I can't think you can't think of anyone who would give it in Queensland for that. But some GP practice nurses are okay with it. But you've got to work out which ones are, and there's not that many. There are minority… You think about it in hospital, they just about panic when they have to give subcutaneous methotrexate and hospital, purple gowns come out everywhere, purple gloves come out everywhere. They've got cytotoxic precaution signs up, which is completely unnecessary…”* **(Rheumatologist, public practice, Queensland, 16-20 years of experience)**  *“… I think there are some logistical barriers. Realistically, even something as simple as managing sharps and I think is not straightforward. Educating patients, regarding it, even though I think there are some reasonable resources out there that's still an extra step. And we don't have the same choice of devices that we would have with biologics. And I think that for various reasons, the administration of sort of biologic agents is better supported than administration of subcutaneous methotrexate. … I think they're fairly clear and obvious reasons why that's the case. Financial. …, I think that's those are probably the biggest barriers.”* **(Rheumatologist, public practice, Victoria, 5-10 years of experience)**  *“…there can be issues with them getting it. The idea of some parenteral methotrexate point of view was that when people were swapping over as well because they would just realize that the other formulation was no longer being manufactured, provided or whatever. So then, … quite a lot of increased demand for Trexject and maybe that's resulted in some shortage. I'm not sure what happened there, but I know there has been issues with getting it at the pharmacy level on occasions.”* **(Rheumatologist, both private and public practice, South Australia, 16-20 years of experience)** |
|  | The availability of pre-filled MTX syringes since 2017 was perceived as an appealing solution to overcome logistical barriers and encourage the consideration of parenteral MTX as a viable treatment option. Further improvements in the design of the injections were suggested to improve the acceptability of the treatment among patients, in which the autoinjectors style of injection perceived as the most preferred type of injection device by the patients. In contrast, injection from a vial was thought to be the most disagreeable method to the patient. The quality of the needle also had a profound effect on the patient’s injection experience, with a sharper needle resulting in less pain during the injection procedure. | *“…I think it [pre-filled syringe] is a lot more attractive. … I'm used to find telling the patients about getting the vial and only drawing as much [they needed], they [the patients] are getting quite stressed about it and some of them are a little bit reticent… I'd be a bit worried about them getting the dosage wrong. And there's a lot of wastage and you know that in things. So, whereas now, I think with a prefilled syringe, I think they come from seven point five to 25 mg dosages. And I actually have a little demonstrator pack in my room, so I'll show them [the patients] …”* **(Rheumatologist, public practice, Victoria, 5-10 years of experience)**  *“…the most common one used nowadays is the Trexject formulation, as in the prefilled syringes. I mean, before they become widely available on the PBS… Maybe four years ago, we used to use the 50 mg in two mLs [vial] for an occasional patient, that again added one logistic barrier as in to educate and make the patient feel comfortable, …titrating their own dose……the prefilled syringes gets rid of that one barrier...”* **(Rheumatologist, public practice, Queensland, 16-20 years of experience)**  *“… Sometimes they [the patients] don't know how to dispose of the pens. So, the syringes. So [if] there's a service that would be good, but otherwise it's preloaded. Someone told me that needles to be blunt…Whether maybe it's the needles, not as fine as we'd like it to be. If it was like a diabetic needle, very small and fine. But I think that would be good as well. Or it comes in a pen or in the autoinjector.”* **(Rheumatologist, private practice, Western Australia, 11-15 years of experience)**  *“… I think the other barrier is that I believe that when you take the injection using the insulins needle from a 50mg vials, it's a more painless procedure because the needles are much higher quality than the Trexject injection, that has a needle that's not as fine as it is.”* **(Rheumatologist, public practice, South Australia, 11-15 years of experience)** |
|  | Although most participants acknowledged that pre-filled methotrexate syringe could facilitate the use of parenteral methotrexate in patients, one pharmacist participant suggested that patients’ outcomes could be further enhanced through sufficient counselling and support in the community setting. And one rheumatologist participant thought the pharmacist would not have the time discussing with patients about parenteral methotrexate in the community settings. | *“… Increased access to the injectable means that supply is more ubiquitous when supply becomes more ubiquitous through community channels, counselling [about parenteral methotrexate] drops. I mean that counselling is not always good in community settings. So it is that basis. So, I think a general lack of understanding with regards to injectable therapies. And that translates into poor outcomes...”* **(Pharmacist, private practice, South Australia, 11–15 years of experience)**  *“… I think if my patient came to me and said, well, my pharmacist said, I should start this medicine [parenteral methotrexate]. I think that would, …be potentially problematic in on a few different ways. But also, … my patients in their two minutes counselling with [pharmacist] never walk away with that discussion, either. So I just don't know how that would work… I'm not to say that that they couldn't be better supports for subcutaneous methotrexate. Ideally, I would make it happen and then give him a card and they'd call someone they call a number and nurse will come around and show them how to inject subcutaneous methotrexate, do the first one at their home. Like the support program and that biologic agent up there. … ideally, that would happen. But, I think in practice that hasn't happened yet.”* **(Rheumatologist, public practice, Victoria, 5-10 years of experience)** |
|  | Time constraints were a barrier reported in most HCPs due to the time needed for education and the availability of a nurse was an enabler. Furthermore, the assistance from general practitioners, pharmacists, nurses, and patient support program were also suggested to be beneficial for the implementation of parenteral methotrexate. | *“…the big thing is education on how to self-inject, and dispose the needle, that's the main thing. I feel like I don't have time in my consult to provide that. In the times that I've had to do that, I've had to book a separate consult to provide that education. I'm always more comfortable when we're got our in-house educator because I know that they're going to be talking to the patients the right thing, but that's the main thing. The education at the time of learning to inject, after that, that's fine.”* **(Rheumatologist, private practice, New South Wales, 5-10 years of experience)**  *“… It could be the fact that it's a little bit more time to explain things to the patients. They [rheumatologists in private settings] don't have time in private practice, ...”* **(Rheumatologist, both private and public practice, South Australia, 16-20 years of experience)**  *“Whereas if they go into a random community pharmacy for a Trexject prescription and the pharmacist isn't overly familiar with it or doesn't completely understand it at all or doesn't have the time to actually sit down and talk to the patient through these things and then there's challenges again in the system.”* **(Pharmacist, private practice, South Australia, <5 years of experience)** |
|  | Most of the resources used by participants were available online, including those provided by organisations such as Arthritis Australia and Creaky Joints. In addition to these resources, several services were identified as beneficial for supporting the use of parenteral methotrexate. Nurse participants reported that the rheumatology clinic at the hospital provided a triage line service, which could address any concerns that patients may have about parenteral methotrexate. Furthermore, some participants suggested that a specialised pharmacy program could enhance the use of parenteral methotrexate in patients. Such a program could involve liaising with rheumatologists to ensure ongoing supply of injections, counselling patients, monitoring safety, and customising the methotrexate injection to meet individual patient requests. | *“…we often will send them and will give them a hyperlink, which has the links to the YouTube video and the ARA has the YouTube of how to actually give it to yourself. And we also have the NPS like form, which is the methotrexate shared care form, which doesn't differentiate whether it's oral or subcutaneous.”* **(Rheumatologist, public practice, Queensland, 16-20 years of experience)**  *“And they [X Pharmacy] would send this [methotrexate injection] to my patients by courier… And they provided all sorts of support and fabulous. They rang up my patients to check that things were going well. They let me know when there were concerns. They provided them with sharps bins... They [ X Pharmacy] would send a pharmacist to the patient's house… So they [X Pharmacy] used to provide injection training as well… The cost was extremely competitive for my patients...”* **(Rheumatologist, private practice, South Australia, 5-10 years of experience)**  *“I run the telephone triage service for the two and a half thousand patient population, rheumatology patients at [X Hospital]. And so, anything urgent is responded to probably at the most within about four hours. If I'm in clinic and they left a message on my phone, I can get back to them within a day.”* **(Nurse, public practice, South Australia, >20 years of experience)**  *“ARA, Creaky Joints, Arthritis Australia. And lots of physios as well, physio input, and, we have a new physio psychology clinic opening up to patients to self-measures. So, at the ARA conference a couple of weeks ago, there was a heavy focus on patient self-measures. So, all about gentle impact exercise, walking, Pilates, yoga and yin-yoga. So all of those joints strength for the exercises. So, we always had that discussion with patients around self-measures and quit smoking…”* **(Nurse, public practice, South Australia, >20 years of experience)**  *“One is the quantity that we dispense, we dispense six syringes each time, so there's a six-week supply for the same cost as a commercially available four-week supply. There are slight differences in the composition of the medicine in the syringe. So, we have one product that is purely methotrexate. One product that is methotrexate diluted with sodium chloride. There are no other additives, which we have had some patients who've used both our products and commercially available, and they have mentioned that our products seem to suit them better. … so the dose can be different because the dose is compounded specifically for the patient, so if the patient needs a twelve point five mg dose, we compounded or X pharmacy compounds it as a twelve point five mg prefilled syringe. So, the safety precautions there, having the accurate dose.”* **(Pharmacist, private practice, New South Wales, 16-20 years of experience)** |
|  | Specialised pharmacy program is available to service parenteral methotrexate users; however, the service was hindered by Coronavirus Disease 2019 (COVID-19) pandemic, geography barrier, insufficient clinical information, and protocol restrictions within residential aged care facility. | *“I don't know if it [COVID pandemic] actually reduced our face to face, but it certainly added its complexities in terms of lockdowns and when people just being generally concerned, …, they're on an immunosuppressant medicine. They know that when they get sick, they get to hold their doses. But there was just that extra layer of concern. As a patient, I think taking that kind of medicine and living with rheumatoid arthritis in that risk of COVID. And I think as well when we started getting the COVID vaccine, I was out of working full time by that point. But when we started, yes, getting COVID vaccinations and all of that just adds a whole another layer of complexity and discussion and education that people are asking for. They need more information from us as pharmacists.”* **(Pharmacist, private practice, South Australia, 11–15 years of experience)**  *“…every aged care facility … have their own regulations and protocols and processes like we did serve a patient who was in the community and then got transferred into an aged care facility, and there was significant barriers around supplying the medicine into the facility, getting it to the right spot and … it wasn't necessarily a regular nurse who was there every day of the week, or who cared for that patient consistently when they were in the facility. So when that little package arrives, that contains the patient's methotrexate, depending on who opens that you don't know where ends up or if they know what to do with that. And we had there was some prescribing challenges as well in terms of getting it on the resident’s medication chart for the nurse to then administer it. And if the doctor accidentally writes, IM, but we've supplied a subcut needle, there is challenges there as well. So what does the nurse do to administer IM but we supply it as subcut because that's what the rheumatologist is prescribed versus what the GP charted on the resident's medication chart. So there was risk of medication error there…, as well as the barriers in terms of the patient continuing to have access to the medicine.”* **(Pharmacist, private practice, South Australia, <5 years of experience)**  *“… that's always a barrier in pharmacy or where pharmacy doesn't quite nail it, it is that we don't have access to the clinical picture. So, when we were providing this program, we could follow up and talk to the patient and stuff. But I've got no idea what the clinical results are. And if the methotrexate is actually working on the supply side, except if the patient tells me that I think it's better…”* **(Pharmacist, private practice, South Australia, <5 years of experience)**  *“…we don't see much about patients. We only talk to them on the phone and that is one potential barrier in communication because there's no body language involved...”* **(Pharmacist, private practice, New South Wales, 16-20 years of experience)** |
|  | General practitioners, pharmacists, nurses, as well as the patient support program were perceived to be beneficial for the implementation of parenteral methotrexate. | *“I have a full-time nurse who largely deals with biologic patients, but also does the educational back up, education on subcutaneous methotrexate, so that's not everywhere has that [service], but we have that. So, and in terms of community education support, I'm not aware of any specific services in the community, but often our nurse access like the central point for a patient to contact…”* **(Rheumatologist, public practice, Queensland, 16-20 years of experience)**  *“… there's not much support around for methotrexate injections, whereas with the biological companies, they have a great nurse program whereby patients enrol, a nurse calls them if there's problems, and then comes to the house, gives them an injection, trains them up, whereas that kind of support doesn't exist for methotrexate. It's probably because the markets … not getting enough money... They would not get that funding to create such a program. So therefore, I rely on the GP practice to do that…I think having this support hotline will be good. So, when the patient is given a script, I can give them a pamphlet with a phone number on it…similar to these support programs of the biological drugs.”* **(Rheumatologist, private practice, Western Australia, 11-15 years of experience)**  *“[GPs are] Very supportive…So I asked the GPs for assistance, and I offer the patients the option of attending my rooms at 8:30 any morning for assistance in injection training. So GPs, the general practice nurses are critical as well. I think the pharmacists could play an important role,...”* **(Rheumatologist, private practice, South Australia, 5-10 years of experience)** |
